# Intra-Individual Variation and Reliability of Biomarkers of the Antioxidant Defense System by Considering Dietary and Lifestyle Factors in Premenopausal Women

**DOI:** 10.3390/antiox10030448

**Published:** 2021-03-13

**Authors:** Alexandra Jungert, Jan Frank

**Affiliations:** 1 Institute of Nutritional Science, Justus Liebig University, Goethestrasse 55, D-35390 Giessen, Germany; 2 Institute of Nutritional Sciences, University of Hohenheim, Garbenstrasse 28, D-70599 Stuttgart, Germany; jan.frank@nutres.de

**Keywords:** reliability, intra-individual variance, antioxidants, vitamins, coenzyme Q10, premenopausal women

## Abstract

Epidemiological studies frequently rely on a single biomarker measurement to assess the relationship between antioxidant status and diseases. This bears an inherent risk for misclassification, if the respective biomarker has a high intra-individual variability. The present study investigates the intra-individual variation and reliability of enzymatic and non-enzymatic biomarkers of the antioxidant system in premenopausal women. Forty-four apparently healthy females provided three consecutive fasting blood samples in a four-week rhythm. Analyzed blood biomarkers included Trolox equivalent antioxidant capacity (TEAC), catalase, glutathione peroxidase, glutathione, vitamin C, bilirubin, uric acid, coenzyme Q10, tocopherols, carotenoids and retinol. Intra- and inter-individual variances for each biomarker were estimated before and after adjusting for relevant influencing factors, such as diet, lifestyle and use of contraceptives. Intraclass correlation coefficient (ICC), index of individuality, reference change value and number of measurements needed to confine attenuation in regression coefficients were calculated. Except for glutathione and TEAC, all biomarkers showed a crude ICC ≥ 0.50 and a high degree of individuality indicating that the reference change value is more appropriate than population-based reference values to scrutinize and classify intra-individual changes. Apart from glutathione and TEAC, between 1 and 9 measurements were necessary to reduce attenuation in regression coefficients to 10%. The results indicate that the majority of the assessed biomarkers have a fair to very good reliability in healthy premenopausal women, except for glutathione and TEAC. To assess the status of the antioxidant system, the use of multiple measurements and biomarkers is recommended.

## 1. Introduction

Chronic diseases, such as cancer and cardiovascular diseases, as well as endometriosis and infertility, are linked to a pro-oxidant metabolic state [1,2,3,4,5]. The term ‘oxidative stress’ is used to describe an imbalance between the production of reactive oxygen species (ROS) and their elimination by antioxidant defense or repair mechanisms [6,7,8,9]. The antioxidant defense system comprises enzymatic and non-enzymatic antioxidants that operate at different levels of ROS elimination and thus counteract a disproportionate ROS formation. Superoxide dismutase, glutathione peroxidase (GPx) and catalase (CAT) are enzymatic antioxidants [10], whereas bilirubin, uric acid, glutathione, ascorbic acid, carotenoids, tocopherols and coenzyme Q10 are non-enzymatic small-molecule antioxidants [11,12].

Although several studies investigated the relevance of antioxidant blood biomarkers in the prevention, pathogenesis and therapy of diseases [13,14,15,16,17], little is known about intra-individual variation of these biomarkers in healthy subjects, especially in women of childbearing age [18]. Epidemiological studies often rely on a single measurement of one biomarker [19,20] with inherent risk for misclassification of the long-term biomarker status as well as biomarker-disease associations, if the respective biomarker has a high intra-individual variability.

Data on intra-individual variation and/or the intraclass correlation coefficient (ICC) as indicator of relative reliability were reported for some blood antioxidant biomarkers [18,19,20,21,22,23,24,25,26]. However, several previous reliability studies represent secondary analyses, gave no information on health status of the subjects, restricted fruit and vegetable consumption, included only subjects with unchanged vitamin intake, included supplement users, used non-fasting blood samples or investigated predominantly non-enzymatic biomarkers with two measuring points several years apart. Furthermore, adjustments for potential influencing factors, such as diet, body mass, physical activity, use of hormones or supplements or sleeping duration were rarely performed. That these factors can have a substantial influence on biomarkers of the antioxidant defense system has been demonstrated [27,28,29,30]. Biomarkers may react very sensitively to changes in endogenous and exogenous factors and thus may not be good biomarkers when only single measurements are performed [31].

Previous studies indicated that biomarkers of oxidative stress are less reliable in females compared to males [21]. Intra-individual variability may be even higher in premenopausal females due to changes during menstrual cycle [32,33]. In order to use antioxidant biomarkers for epidemiological studies, the extent of intra-individual variation and the reliability of the respective biomarkers as well as the degree of individuality and the resulting practicality of population-based reference values require scrutiny.

The present study aims to scrutinize the intra-individual variation in and reliability of enzymatic and non-enzymatic biomarkers of the antioxidant defense system in premenopausal women over eight weeks of follow-up by considering relevant confounders, such as diet, lifestyle, use of contraceptives and sleep duration.

## 2. Methods

### 2.1. Ethical Standards

This study was conducted according to the guidelines laid down in the Declaration of Helsinki and approval was granted by the Ethical Committee of the Faculty of Medicine at the Justus Liebig University of Giessen, Germany (Project identification code AZ 201/17). Written informed consent was obtained from all subjects.

### 2.2. Study Design and Population

This longitudinal study was conducted at the Institute of Nutritional Science at the Justus Liebig University of Giessen and the blood donation center at the University Hospital of Giessen and Marburg, Germany. Recruitment was performed between January and April 2018 via social media, email, notices, personal contacts and oral presentations by staff and students of the Institute of Nutritional Science, Justus Liebig University of Giessen, Germany. The investigation period ranged between March and June 2018. The subjects visited the blood donation center at the University Hospital of Giessen and Marburg, Germany, three times at intervals of 28 ± 4 days to give a blood sample at each visit. The time interval was chosen to limit the impact of the menstrual cycle and to reflect an assessment approach that is likely to be performed in clinical or research settings. Subjects had to fill out a self-administered questionnaire and a three-day estimated dietary record covering the last three days before each blood sampling. Furthermore, body temperature was measured by an ear thermometer (Braun Welch Allyn, Inc., New York, NY, USA) before blood sampling to exclude the presence of fever.

Inclusion criteria were female sex, age between 20 and 35 years, non-smoker for at least six months before start of the study and good comprehension of the German language. Exclusion criteria were male sex, peri- or postmenopausal state, body mass index (BMI) ≥ 30.0 kg/m^2^, pregnancy or lactation in the last 12 weeks before the study, oophorectomy, hysterectomy, organ transplantation, life-time diagnosis of cancer as well as diagnosed chronic diseases, such as gout, rheumatism, Parkinson’s disease, inflammatory bowel diseases, asthma, obstructive pulmonary disease, thyroid diseases and chronic infectious disease. Subjects who suffered from influenza or tropical infectious disease four weeks before and during the study period were excluded. Use of allopurinol, diuretics, cytostatics, statins, antidepressants, anticoagulants, antacids, antibiotics, corticoids and drugs against rheumatism two months before and during the study were not permitted. Intake of systemic analgesics, such as aspirin, ibuprofen or paracetamol and antihistamines was not allowed within the two months before baseline analysis. Non-regular intake of systemic analgesics or antihistamines after completing the baseline analysis was tolerated and considered via sensitivity analysis (for further explanations see “Statistical analyses”). Further exclusion criteria comprised eating disorder or addiction disorder within 12 months before the study, intentional fasting or energy restriction in the last four weeks before and during the study in such a manner that the diet differs from habitual eating behavior of the individual. Subjects completed the German Eating Disorder Examination-Questionnaire, which is based on a semi-structured interview and contains 22 items to identify specific eating disorder psychopathology [34]. Finally, the use of dietary supplements, such as vitamins A, C and E, carotenoids as well as multi-vitamin supplements, were not permitted four weeks before and during the study. Intake of fortified foods was not restricted.

Subjects were asked to follow their habitual diets during the study period and instructed to abstain from alcohol consumption 24 h and from intense physical activity 12 h before the physical examinations.

In total, 174 subjects were interested in participating in the study. Of the 72 subjects, who were deemed eligible to take part in the study and provided written informed consent to participate, 61, 48 and 46 completed the baseline analysis, first follow-up and second follow-up, respectively. Of the 46 subjects who completed the study, one blood sample showed signs of lipemia and another an intense yellow color; thus, both samples and in consequence both subjects were not considered in the analysis, leaving 44 subjects of whom the majority were white (>90%). The sample size for measuring the enzymatic biomarkers was slightly reduced due to atypical hemoglobin values in four erythrocyte lysate samples, leaving 40 subjects with three available measurements. The flow chart of the study is illustrated in Supplementary Figure S1.

### 2.3. Biochemical Analyses

A fasting blood sample of around 40 mL from a peripheral vein was collected from the participants by a qualified phlebotomist at the three times of data collection in the morning hours (07:50–09:30 a.m.). Subjects were asked to fast and to drink no liquids apart from water for at least 12 h. Before blood sampling, subjects rested on an examination bed in a supine position for at least 10 min. BD Vacutainer^®^ systems were used, which comprised a 21-gauge needle, spray-coated clotting agents for serum samples and spray-coated lithium heparin or K2 ethylenediaminetetraacetic acid (EDTA) for plasma samples. After collection, tubes were gently inverted. Serum samples were stored at room temperature and plasma tubes at 7–10 °C under exclusion of light until they were transported to the Institute of Nutritional Science. Blood samples showed no visual evidence of hemolysis and were centrifuged at around 1400× *g* for 10 min at 4 °C. Plasma and serum were immediately aliquoted into coded freezing tubes. Erythrocytes were washed three times using 0.85% sodium chloride solution to remove platelet-rich plasma, buffy coat layer and leukocytes. Lysates of erythrocytes were prepared comprising 1 mL washed erythrocytes and 4.5 mL 0.85% sodium chloride solution. For vitamin C determination, heparin plasma samples were acidified with trichloroacetic acid before freezing to precipitate proteins and stabilize vitamin C. For glutathione determination, whole blood samples were deproteinized with 5-sulphosalicylic acid, centrifuged and supernatant solutions were frozen. Blood samples were stored at −70 to −80 °C until analysis.

Immediately after blood collection, standard hematological parameters were measured on Sysmex KX-21N (Sysmex, Kobe, Japan) at the blood donation center using EDTA blood samples. The measurements of Trolox equivalent antioxidant capacity (TEAC), antioxidant enzymes, glutathione and vitamin C were performed by qualified staff of the Institute of Nutritional Science at the Justus Liebig University, Giessen, Germany. Serum concentrations of uric acid, cholesterol, triacylglycerol, coenzyme Q10 and high-sensitive C-reactive protein (hs-CRP) were measured in the medical laboratory Dres. med. Walther, Weindel and Colleagues in Frankfurt, Germany. Plasma concentrations of tocopherols, carotenoids and retinol were determined by qualified staff of the Institute of Nutritional Sciences at the University of Hohenheim, Germany. Frozen serum and plasma samples were transported with dry ice to their destination. Biochemical parameters were measured at least in duplicates (batch analysis) and the mean value was calculated except for hs-CRP, which underwent single determination because it was only assessed as control factor to identify subjects suffering from inflammatory conditions. Blood parameters were analyzed within one year after collection. The stability of the antioxidant biomarkers under such freezing conditions was demonstrated in previous studies [18,35,36]. Quality controls were performed by repeated analyses of stored pooled samples and/or internal/external standards. Assessment methods, study location, equipment and devices were constant over the course of the study.

#### 2.3.1. Non-Enzymatic Antioxidants

##### Serum Concentrations of Total Bilirubin

Total bilirubin was assessed by commercial colorimetric diazomethod on Cobas 8000 [37]. In two serum samples, measurements were below the limit of quantification (0.1 mg/dL) and those values were set to the respective limit of quantification before calculation of mean values. The coefficients of variation were ≤2.5% and ≤3.3% with respect to repeatability and intermediate precision using human serum of different concentrations, respectively [37].

##### Serum Concentrations of Uric Acid

A commercial enzymatic colorimetric test on Cobas 8000 was used, in which serum uric acid is cleaved by uricase to form allantoin and hydrogen peroxide [38]. The coefficient of variation (CV) was ≤0.6% and ≤1.3% with respect to repeatability and intermediate precision using human serum of different concentrations, respectively [38].

##### Plasma Concentrations of Vitamin C

Plasma vitamin C was determined by the 2,4-dinitrophenylhydrazine (DNPH) colorimetric method [39,40]. The absorbance of the DNPH derivative was measured at 520 nm (Shimadzu UV-1800, Shimadzu, Kyoto, Japan) with an intra-day CV of ≤4.8% and between-days CV of 3.0%.

##### Plasma Concentrations of Tocopherols, Retinol and Carotenoids

In lithium heparin plasma, α- and γ-tocopherol, carotenoids (lutein, zeaxanthin, β-cryptoxanthin, lycopene, α-/β-carotene) and retinol were simultaneously measured using a Shimadzu (Kyoto, Japan) Prominence high performance liquid chromatography (HPLC) equipped with a LC-20 AT pump and a RF-10A fluorescence detector and a SPD 20A UV-vis detector as described previously [41,42]. Using pooled plasma, inter-batch CV were 3.1% for α-carotene, 3.7% for β-carotene, 3.9% for lutein, 6.7% for zeaxanthin, 3.4% for β-cryptoxanthin, 7.6% for lycopene, 4.1% for γ-tocopherol and 6.3% for α-tocopherol and 3.7% for retinol [41].

##### Serum Concentrations of Coenzyme Q10/Ubiquinone

Coenzyme Q10 was assessed in oxidized form, that is, ubiquinone. Ubiquinol in the sample was oxidized to ubiquinone, separated from lipophilic proteins and the concentration measured by HPLC with UV detection using a commercial Chromsystems kit [43]. Intra-assay CV was ≤4.8% and inter-assay CV was ≤5.8% using human plasma of different concentrations, respectively [43].

##### Whole Blood Concentrations of Total Glutathione

The method to measure total glutathione in venous EDTA blood samples was based on the enzymatic cycling assay first described by Tietze [44] and modified by Becker et al. [45]. Ellman’s reagent, 5,5′-dithiobis(2-nitrobenzoic acid) (DTNB), is reduced to 2-nitro-5-thiobenzoic acid (TNB) by glutathione. The formed glutathione disulfide is recycled to glutathione by glutathione reductase using reduced nicotinamide adenine dinucleotide phosphate (NADPH) as cofactor. Reduced glutathione reacts with DTNB again. The absorbance of TNB at 412 nm (Shimadzu UV-160A, Shimadzu, Kyoto, Japan) is proportional to the concentration of glutathione. Using pooled plasma, the intra-day and inter-day CV was ≤4.0% and 1.9%, respectively.

##### Plasma Trolox Equivalent Antioxidant Capacity (TEAC)/Total Antioxidant Status

The principle behind the assessment of TEAC is based on the inhibition of the 2,2-azino-bis-3-ethylbensthiazoline-6-sulfonic acid (ABTS^+^) radical formation by antioxidants [46]. Plasma TEAC was determined by photometric detection of the formation of the ABTS^+^ radical at 600 nm (Shimadzu UV-160A, Shimadzu, Kyoto, Japan) in comparison to Trolox. The latter is a water-soluble analogue of α-tocopherol [46]. Thus, the results have to be interpreted as the reactivity relative to 1.0 mmol Trolox/L. Using pooled plasma, the intra-day and inter-day CV was ≤1.8% and 6.5%, respectively.

#### 2.3.2. Antioxidant Enzyme Activities in Erythrocytes

Activities of the antioxidant enzymes CAT and GPx were measured in erythrocyte lysates by photometric detection. Enzyme activities are expressed as units per 1 g hemoglobin (Hb). Drabkin’s reagent was used for the determination of Hb in erythrocyte lysate. Absorption was measured at 540 nm (Shimadzu UV-1800, Shimadzu, Kyoto, Japan).

The activity of erythrocyte GPx (eGPx) was measured based on a coupled assay by the method of Paglia and Valentine [47]. GPx catalyzes the reduction of hydrogen peroxides by the oxidation of glutathione. The latter is reduced by glutathione reductase and NADPH. The decrease in the absorbance of NADPH at 340 nm was measured (Shimadzu UV-1800, Shimadzu, Kyoto, Japan). The reaction was initiated with addition of tert-butyl hydroperoxide. Experiments were performed at 37 °C in Tris buffer (pH 8.0). Using pooled washed erythrocytes, the intra-day and inter-day CV was ≤3.7% and 6.6%, respectively.

The activity of erythrocyte CAT (eCAT) was assessed by measuring the elimination of defined quantities of hydrogen peroxide within one minute by CAT available in the erythrocyte lysate. After one minute incubation with hydrogen peroxide, ammonium molybdate solution was added to stop the reaction. The remaining hydrogen peroxide within the sample reacted with hydrogen peroxide and formed a yellow color complex, which was measured at 405 nm after 5 min (Shimadzu UV 1800, Shimadzu, Kyoto, Japan). Experiments were performed at 37 °C in di-sodium hydrogen phosphate and di-potassium hydrogen phosphate trihydrate buffer (pH 7.4). This assay was based on the methods by Aebi [48] and Goth [49]. Using pooled washed erythrocytes, the intra-day and inter-day CV was ≤8.0% and 7.4%, respectively.

#### 2.3.3. Associated Factors

##### Serum Concentrations of Cholesterol

Serum cholesterol was measured by a commercial enzymatic colorimetric test on Cobas 8000 according to manufacturer’s instructions [50]. The CV was 0.6% and ≤1.6% with respect to repeatability and intermediate precision using human serum of different concentrations, respectively [50].

##### Serum Concentrations of High-Sensitive C-Reactive Protein (hs-CRP)

Following the manufacturer’s instructions, hs-CRP was measured on Cobas 6000 using a particle-enhanced (latex) immunoturbidimetric assay [51]. The CV was ≤1.6% and ≤8.4% with respect to repeatability and intermediate precision using human serum of different concentrations, respectively [51].

### 2.4. Anthropometric Data

After bladder emptying, body mass and height of the participants were measured while standing in an upright position using a digital calibrated scale with stadiometer (SECA, Hamburg, Germany) in light clothing without shoes to the nearest 0.1 kg and 0.1 cm, respectively, before each blood sampling. The average of the three body height measurements was used for the subsequent BMI calculation. BMI (kg/m^2^) was calculated by dividing body mass in kg by the square of body height in meter.

### 2.5. Data on Education, Health Status, Lifestyle, Use of Drugs and Supplements

Before each physical examination, subjects completed a self-administered questionnaire on education, smoking behavior, sleep duration, diseases, vaccinations as well as use of medicine and supplements.

Subjects self-rated their wellbeing as very good, good, fair, poor or feeling stressed. Sleep duration was assessed by asking the subjects on their time falling asleep at night and waking up in the morning covering the last three days before blood sampling. In addition, subjects were asked on their usual sleep duration during day and night time. Based on the three reported days, the average sleep duration was calculated in min/d. One subject reported in one follow-up the requested sleeping time only for the first day. Thus, the average of the reported time and the usual sleep duration was calculated. In addition, weekly physical activity pattern was assessed by asking the subjects how much time they spend on physical activity including sports activities, household chores and occupation.

The physical activity level (PAL) was calculated as the ratio of the resting metabolic rate (RMR) to the total energy expenditure. RMR was calculated using the equation published by Müller et al. [52], whereas energy expenditures of the different activities and non-activities (including different sports, occupation, household chores, sleep) were calculated using metabolic equivalents (MET) published in the 2011 updated Compendium of Physical Activities [53]. MET were multiplied by the RMR in kcal/min and the time spend on the respective activity in min/d. For the remaining time of the day which was not spend on the above mentioned activities and non-activities, a MET factor of 1.4 was assumed reflecting light effort activities, such as sitting, studying, reading and writing [53].

### 2.6. Data on Hormonal Status

A self-administered questionnaire was handed out to the participants including questions on the use of contraceptives, the day of the beginning of the last menstrual period, the age at menarche and the habitual duration of the menstrual cycle categorized in <25 days, between 25 and 35 days and >35 days.

### 2.7. Data on Dietary Factors

Three times, a self-administered estimated dietary record covering the last three consecutive days before blood sampling was handed out to the participants. The participants estimated the portion sizes by using household measures. Examples of typical food portion sizes were included within the description provided with the dietary record. Each participant was instructed by a trained nutritionist. Mean dietary intake of energy and nutrients were calculated using the software DGExpert, version 1.9.2, with the German Nutrient Database version 3.02 (Max Rubner-Institute, Federal Research Institute of Nutrition and Food, Karlsruhe, Germany). Furthermore, participants were asked by a questionnaire, whether they follow a specific diet (e.g., vegetarian, vegan).

The magnitude of under- and over-reporting was assessed for each point of data collection on individual level by the revised approach of Goldberg et al. [54] described by Black [55]. MET are defined in the Compendium of Physical Activities as “the ratio of a person’s working metabolic rate relative to their resting metabolic rate” [53]. Thus, we replaced basal metabolic rate by RMR in the equation by Black [55]. The mean PAL of the study population was 1.58, 1.60 and 1.63 at baseline, first and second follow-up, respectively. We used the CV suggested by Black [55] for repeated metabolic rate measurements (8.5%), inter-individual variation in PAL (15%) and intra-individual variation in energy intake (23%). Overall, 20%, 16% and 9% of the subjects were identified as low-energy intake reporter at baseline, first and second follow-up, respectively. No subject was identified as high-energy reporter.

### 2.8. Statistical Analyses

Statistical analyses were performed with SPSS version 26 and R versions 3.5.1 and 3.6.3 using the packages corrplot [56], version 0.84, ggplot2 [57], version 3.3.0, and lme4 [58], version 1.1-23, with support of R packages car [59], version 3.0-7, and lattice [60], version 0.20-41. Results were considered statistically significant at a *p* value of <0.05.

Power calculation was a priori determined by R package ICC.Sample.Size [61]. Under the assumption of a probability of type 1 error of 5% and a power of at least 80%, 36 subjects were necessary to detect an ICC of 0.30 provided the availability of three repeated measurements. Assuming a dropout rate of 20%, a minimum sample size of 44 subjects was calculated.

Because several variables were not normally distributed (tested by Shapiro-Wilk test), descriptive data are expressed as median and interquartile range (25 to 75 percentiles). Differences in antioxidant biomarkers across measurement time points were tested using the Friedman test and pairwise comparisons using the Wilcoxon signed-rank test with Bonferroni adjustment were performed if the overall test was significant. A correlation matrix based on Spearman’s correlation analysis was illustrated to investigate the interrelations among the biomarkers at baseline.

As recommended by Braga and Panteghini [62], test for outliers among intra-individual variances was performed via Cochran’s test and the Reed’s criterion was used to identify outliers among the mean biomarker values of subjects. Subjects who were identified as outliers were excluded from the following analyses. Based on the results of the Shapiro-Wilk test, all biomarkers, except bilirubin, eCAT, glutathione and TEAC, underwent logarithmical transformation. The hypothesis of normal distribution of the transformed and, in case of eCAT, glutathione and TEAC, non-transformed biomarker values was accepted in most subjects according to Shapiro-Wilk test, apart from bilirubin.

To scrutinize the reliability of the biomarker measurements, the ICC was calculated as inter-individual variance divided by the total variance (i.e., sum of intra- and inter-individual variance) [23]. Both variance components were estimated by the restricted maximum likelihood method. At first, a one-way random effect model was created for each biomarker with the respective biomarker as dependent variable and subject ID as random effect. Following, the ICC was calculated after adjusting for relevant covariates, such as time elapsed since the first day of the last menstrual period (d), BMI (kg/m^2^), fasting duration (h), sleep duration of the last night (h), use of contraceptives (no vs. yes), serum cholesterol concentration (mmol/L) and PAL. To take into account changes in biomarkers driven by variations in dietary intake, energy intake (MJ/d) or, if applicable, nutrient intake levels (i.e., purine intake (mg/d) for serum uric acid, vitamin C intake (mg/d) for plasma vitamin C, tocopherol equivalents intake (mg/d) for both plasma tocopherol biomarkers, retinol equivalents intake (mg/d) for plasma retinol and β-carotene intake (mg/d) for plasma carotenoid biomarkers) were included in the model. The covariates were included as fixed effects in the linear mixed-effects model and data from all three measuring time points were considered. Age was not included as fixed effect because of the small age range.

There is no uniform agreement on the cut-off values and interpretation of ICC. For blood biomarkers, the ICC can be judged as follows: ICC ≥ 0.75 = very good reliability, ICC 0.51–0.74 = good reliability, ICC 0.40–0.50 = fair reliability and ICC < 0.40 = poor reliability [24]. According to Kotsopoulos et al. [24], an ICC ≥ 0.40 allows an interpretation of a biomarker on a long-term perspective from a single measurement.

The total intra- (CV_T_) and inter-individual (CV_G_) coefficients of variation were calculated. For log-transformed data, the formula of lognormal distribution was applied to give an approximation for the variance components on the original scale of the biomarker [63]. The index of individuality (II) represents the ratio of the total intra-individual to inter-individual variation, and an II > 1.4 denotes low individuality [64]. In the case of an II < 0.6, the reference change value (RCV) seems more appropriate than population-based reference values [64]. The symmetrical RCV was calculated by the formula: RCV = [Z × 2^0.5^ × CV_T_^2^] [62]. For log-transformed data, the asymmetrical up and down RCV were calculated using the lognormal approach described by Fokkema et al. [63]:

RCV = [exp (Z × 2^0.5^ × σ) − 1] × 100 (1)

σ = [ln((CV_T_)^2^ + 1)]^0.5^ (2)

CV_T_ = [exp(σ^2^) − 1]^0.5^ (3)

In this formula, Z = ±1.96 and thus determines the RCV bidirectionally at 95% probability level [64]. RCV is defined as percentage change in serial measurements indicating significant changes in the respective biomarker [62].

Finally, the number of measurements needed to confine the attenuation to 10% and 20% of the ‘true’ regression coefficient with the biomarker as independent variable was calculated [19,23] by the formula *n_β_* = [*P*/(1 − *P*)][*S_w_*^2^/*S_b_*^2^] [65]. *S_w_*^2^ and *S_b_*^2^ denote intra- and inter-individual variances, respectively; and *P* = 1 minus the maximum allowed attenuation. Analogous to Block et al. [23], the number of required measurements was rounded up to the next integer if more than 0.20 higher than the lower integer.

Residual distribution from the fitted models were evaluated by qq plots and heavy tailed distributions or outlying residuals were partially noticed. It is well-known that a few outlying values can have a considerable impact on the variance estimates when the sample size is limited [23]. Thus, the 95% confidence intervals (CI) of the ICC were calculated using a bootstrapped distribution of 2000 bootstrap samples. In addition, two sensitivity analyses were performed, whereupon the analyses were repeated after:

1. Model-wise exclusion of subjects with at least one outlying residuum (i.e., >2.0) based on the performed linear mixed-effects models. Exclusions were carried out until no further outlying residuum was detected.
2. Exclusion of subjects who reported the use of contraceptives (*n* = 22), non-regular use of analgesics/antihistamines (*n* = 5), vaccination (*n* = 1), symptoms of abnormal menstrual cycle (*n* = 3) or health problems/restrictions, for example, food intolerances/allergies (*n* = 6) as well as one subject with hs-CRP concentration ≥ 95 nmol/L (≥10 mg/L). Some subjects meet more than one of these exclusion criteria. Due to the reduced sample size, the adjusted ICC was not determined. Before analysis, biomarkers were again checked for outliers via Cochran’s test and Reed’s criterion and residuals were again inspected for outliers (i.e., >2.0).

## 3. Results

The descriptive data of the 44 subjects at each assessment before exclusion of outliers are shown in Table 1. The subjects had a median age of 23 years (range: 20–30 years). All subjects have obtained a high school graduation and 91% of the subjects were students. According to auricular temperature measurements and questionnaire data, none of the subjects suffered from fever in the course of the study. In each assessment period, the majority of the subjects rated their wellbeing as *fair*, *good* or *very good* (≥89%), while ≤11% reported being *often stressed*. No female rated her wellbeing as *bad*.

Age at menarche ranged between 10.0 and 16.5 years. Around 80% of the subjects reported a cycle length between 25 and 35 days. The median time elapsed since the first day of the last menstrual period was equal across assessment periods (tested via Friedman test; *p* = 0.300). The majority of women who used contraceptives reported using the combined hormonal pill with estrogen and gestagen (82%), while four subjects used vaginal rings, intra-uterine contraceptive devices (coil) or a hormonal pill that contained only gestagen.

For several antioxidant biomarkers, significant differences across measurement time points were noticed (Table 1). In Spearman’s correlations, significant positive and negative correlations among the biomarkers were found at baseline, whereupon carotenoid biomarkers were particularly positively interlinked with each other and with vitamin C, α-tocopherol, coenzyme Q10 and TEAC (Supplementary Figure S2). Bilirubin, glutathione and eGPx were the only parameters that did not significantly correlate with other antioxidant biomarkers at baseline. In Supplementary Figure S3, mean values of the respective biomarkers and absolute ranges are illustrated for each subject.

Outliers were detected for bilirubin (*n* = 2), γ-tocopherol (*n* = 1), α-carotene (*n* = 4), β-carotene (*n* = 3), lutein (*n* = 1), β-cryptoxanthin (*n* = 3), glutathione (*n* = 1) and TEAC (*n* = 1), and the corresponding subjects were excluded from the following analyses.

The crude and adjusted ICC, intra- and inter-individual CV, II, RCV and the number of measurements needed to confine the attenuation to 10% and 20% of the ‘true’ regression coefficient are shown in Table 2. Crude ICC ranged from 0.000 for plasma TEAC to 0.875 for plasma β-carotene. Except for glutathione and TEAC, all biomarkers showed a crude ICC > 0.50. Adjustments for covariates markedly reduced the ICC of plasma α-tocopherol, plasma lutein, plasma lycopene and serum coenzyme Q10, while the ICC of the other biomarkers was largely unaffected. The lowest inter-individual variations were found for eCAT, glutathione and TEAC. All biomarkers showed a total intra-individual variation below 33%. The lowest intra-individual variations were observed for eCAT and TEAC. The majority of the biomarkers had an II between 0.5 and 1.0 and only glutathione had an II > 1.4. For TEAC, no II could be calculated due to its very low inter-individual variation. Apart from glutathione and TEAC, between 2 and 9 biomarker measurements were necessary to reduce attenuation in regression coefficients <10%, while for <20% attenuation between 1 and 4 measurements were needed.

After excluding subjects with outlying residuals, crude and adjusted ICC of biomarkers remained either unchanged or improved, whereas intra-individual variation, RCV and the number of required measurements to limit the attenuation in regression coefficient were reduced in most biomarkers (Supplementary Table S1). Considerable positive changes in crude ICC (difference ≥+0.100) were found for γ-tocopherol, zeaxanthin, β-cryptoxanthin and glutathione as a result of decreased intra-individual variation rather than increased inter-individual variation.

As illustrated in Table 3, the exclusion of subjects with conditions that may influence biomarker status and of subjects with outlying residuals led to a marked decrease in crude ICC for uric acid, γ-tocopherol and β-cryptoxanthin, whereas the ICC for glutathione improved compared to the results of the first sensitivity analysis (Supplementary Table S1). Still, all biomarkers showed an intra-individual variation below 33% and glutathione, eCAT and TEAC remained the biomarkers with the lowest intra- and inter-individual variations, albeit plasma α-tocopherol exhibited also a low intra-individual variation in the investigated subsample. The II of the majority of the biomarkers was still between 0.5 and 1.0 and even glutathione exhibited an II < 1.4.

## 4. Discussion

The unique feature of the present study is the investigation of the reliability and intra-individual variation of a wide range of biomarkers of the antioxidant defense system in premenopausal women before and after adjusting for relevant influencing factors. To our knowledge, no study so far addressed parameters of intra-individual variability and reliability of eCAT, eGPx, total glutathione, coenzyme Q10 and TEAC in healthy young women. The results may help to distinguish between intra-individual changes in biomarkers of the antioxidant defense system, which are inherent physiological variations, from those caused by interventions or pathological conditions.

This study indicates that the majority of the assessed biomarkers of the antioxidant defense system have a fair to very good reliability but also a high degree of individuality. The latter indicates that the RCV is more appropriate than population-based reference values to scrutinize and classify intra-individual changes in these biomarkers. The highest intra-individual variations are observed for serum bilirubin, plasma γ-tocopherol and some plasma carotenoids. Judged by the crude ICC, plasma α-carotene, plasma β-carotene, serum coenzyme Q10 and eGPx are the most reliable biomarkers, whereas glutathione and TEAC had the poorest reliability owing to their very low inter-individual variance. Controlling for serum cholesterol and other relevant covariates attenuates the ICC for some lipid-related biomarkers. For most biomarkers multiple measurements are required to reduce attenuation in regression coefficients to 10%.

As the investigated biomarkers showed predominantly a good reproducibility, a single measurement of the biomarker allows conclusions on the long-term biomarker status under non-pathological conditions. However, the lower bound of the 95% CI of ICC was partly below the threshold of 0.4. The impact of circadian variations, lifestyle, diet, hormonal changes [32] and/or medications on the biomarker may lead to wrong conclusions about the antioxidant defense system. The observed variation in biomarkers over time may reflect the dynamic of the antioxidant defense system and/or the sensitivity of the biomarkers to changes in endogenous and/or exogenous factors. However, based on the evaluated dietary records, no major changes in dietary intake of selected nutrients and energy intake in the course of the study were found; except for tocopherol intake, which decreased across sampling time points (*p* < 0.05) accompanied by decreasing α-tocopherol concentrations (Table 1). In contrast, PAL slightly increased over time (*p* < 0.05), which can probably be explained by changing weather conditions, such as increased temperatures, and the beginning of the semester sport courses. Biomarkers of which the ICC attenuated after adjusting for influencing factors exhibit a stronger reduction in inter-individual variance than in intra-individual variance. Controlling for these influencing factors in epidemiological studies is necessary [21] when analyzing the association between biomarker status and disease risk as well as changes in the course of aging. Epidemiological studies frequently exhibit a wide time gap between biomarker measurement and clinical disease manifestation. This implies high demands on biomarker measurements especially when the biomarker is analyzed only once.

The reported ICC values in the literature varied greatly. For example, in studies which did not focus on circadian effects or changes within menstrual cycle, the following ICC values were reported: bilirubin 0.4–0.8 [21], uric acid 0.7–1.0 [21], vitamin C 0.4–0.8 [20,23], α- and/or γ-tocopherol 0.1–0.9 [19,23,26], retinol 0.4–0.9 [19,20,26], carotenoids 0.2–0.8 [19,20,23], total antioxidant capacity (equivalent to TEAC) 0.01 [66] and reduced/oxidized glutathione 0.6 [25]. Overall, the ICC values of the present study are in the range of previously reported ICC values, with the exception of glutathione. In this particular study, the ICC for reduced/oxidized glutathione, not total glutathione, was calculated in a population of 12 resistance-trained men with 7 visits within one month [25]. Besides the fact that the available studies differ considerably in their designs, we want to emphasize that ICC and CV are population-specific estimates and thus not pertinent without restrictions to population groups with inherently different levels of heterogeneity [67].

For clinical implications, it is essential to distinguish between a temporary intra-individual change in biomarker status and a change that characterizes a decline or an increase in biomarker status due to intervention or pathological processes [68]. The high degree of individuality observed in the present study implies that there is an increased risk that a measurement result of a biomarker is quite far from the individual homeostatic set point but would be judged as non-pathological as the value may be still within population-based reference values [62,64]. Thus, the RCV may be used to evaluate relative differences in consecutive biomarker measurements instead of general population-based thresholds [64]. As shown in Table 2, biomarkers with relatively high intra-individual CV exhibited higher RCV.

To allow a reliable extrapolation from a single measurement to long-term biomarker status, the inter-individual variance had to be the overriding proportion of the observed total variance in the respective biomarker compared to the intra-individual variance [31]. In the present study, this was particularly the case for α- and β-carotene, while for vitamin C intra- and inter-individual variances comprised about equal proportions of the total variance and inter-individual variance in TEAC went to zero. The low inter-individual CV found for TEAC and glutathione imply that the measurement values are close to the mean value of the population. In a previous study, which reported the intra-/inter-individual variation of 20 apparently healthy subjects based on eight consecutive days, glutathione expressed as µmol/g Hb showed an intra-/inter-individual variation of around 14% and 11%, respectively [69], which is comparable to our results with regard to CV_T_ (10–16%) and slightly higher with regard to CV_G_ (5–9%). In a recent study analyzing the intra- and inter-individual variation and ICC for the total antioxidant capacity, based on two afternoon and three morning collections over two weeks in ten healthy subjects, a very low ICC of 0.01 and an intra-individual variation of 8% was reported [66], similar to our observations. In addition, the total antioxidant capacity was the parameter with the lowest inter-individual variation, albeit inter-individual variation was higher (8%) than in the present study [66]. Because of the differences in study designs and populations, the results are not directly comparable. However, the somewhat higher inter-individual variation in glutathione and total antioxidant capacity might be explained by the fact that females and males were analyzed together in these two studies. TEAC and glutathione may exhibit a higher inter-individual variance in populations with higher heterogeneity in age, sex, body composition, fasting state and especially health status. Nevertheless, the findings for TEAC and glutathione have to be interpreted in the light of the methodological limitations [70,71,72]. TEAC focusses on the scavenging of the non-physiologic ABTS^+^ radical, is not an indicator for a single antioxidant and the results may not reflect an exact anti-oxidant activity [70,72], while glutathione was not differentiated in reduced/oxidized glutathione in the present study.

Excluding subjects with outlying residuals improved the ICC of several parameters but did not substantially impact the parameters with the lowest reliability. A single assessment of a biomarker with poor reliability carries the risk that the observed association between the investigated biomarker status and the respective disease is lower than the actual relation [21,23].

In agreement with previous studies that analyzed micronutrients [19], our results indicate that measurements of antioxidant blood biomarkers have to be obtained more than once to characterize the biomarker status of a subject. Repeated assessments of biomarkers from each subject decrease the impact of intra-individual variability [19]. Alternatively, the sample size could be increased to limit the total variability [19]. The question is, how many measurements from an individual have to be taken to confine the attenuation to 10% and 20% of the ‘true’ regression coefficient. For the majority of the biomarkers, 3 to 5 measurements were enough to adequately address the intra-individual variance within regression analyses. For correlation coefficients, the number of measurements needed for an attenuation can be expected to be lower [19].

Biomarkers of the antioxidant system interact with each other and some antioxidants may function as prooxidants under certain conditions. It is highly questionable, to what extent a highly dynamic system like the antioxidant defense system can be evaluated by means of a single blood sample of one biomarker [24,73]. Even in our relatively homogeneous study population, the intra-individual variances within biomarkers were far from uniform and correlation analysis indicated positive and negative interrelationships among the biomarkers, albeit primarily on a relatively low level. Thus, a single biomarker reflects at best the respective component of the antioxidant defense system but is clearly limited in predicting the state of the entire and highly complex system. Hence, the simultaneous assessment of a set of antioxidant biomarkers, for example by metabolomics approaches, is recommended to assess the status of the antioxidant system in depth. Furthermore, reliability of the biomarkers under disease conditions should be investigated. It was demonstrated that levels of antioxidants could be amplified as well as reduced in pro-oxidant state [8]. The sole consideration of blood antioxidant biomarkers falls too short to reflect a state of oxidative stress. Thus, biomarkers reflecting oxidative processes and arising oxidative damages should be assessed in conjunction with antioxidant biomarkers in different tissues [8].

The present results should be interpreted in the light of the following limitations. The study population is quite homogeneous due to the inclusion criteria and thus the results cannot necessarily be generalized to other study populations. Around 40% of the subjects followed a vegan or vegetarian diet and thus it can be assumed that females with a strong interest in a healthy lifestyle may be particularly interested in participating in this study. Nevertheless, the biomarker status showed a wide range of concentrations and activity levels. The data do not allow for any conclusions regarding the impact of the menstrual cycle on antioxidant biomarkers as the assessment was performed in a four-week rhythm to minimize the impact and data on circulating sexual hormones were not obtained. As the study lasted from March to June, changes in lifestyle due to a seasonal effect cannot be excluded but may be to some extent reflected by the obtained data on dietary intake and physical activity, albeit the data on dietary intake and physical activity were based on self-report. The dietary records data point to the well-known issue of under-reporting, which is often observed for foods rich in sugar and fat rather than vitamins and antioxidants. Therefore, the under-reporting may be of limited concern in the present study, but the data do not allow us to conclude whether over-reporting of so-called ‘healthy’ food has occurred. Moreover, the nutrient database provided no data on the dietary intake of several antioxidants. The observed intra-individual variance may be to some extent attributed to a random error in laboratory performances [21], although daily quality controls in the course of the present study reveal no cause for concern. Analytical imprecision based on quality control materials ranged from 1% to 8% and replicate measurements of each sample counteract a random error due to laboratory assessment. In sensitivity analysis, the power may be insufficient to scrutinize low ICC due to the small sample size. Finally, the results cannot be unrestrictedly extrapolated to studies with longer follow-up periods.

The following strengths of the present study should also be considered—the study was based on a community-dwelling well-described study population of premenopausal women in which enzymatic and non-enzymatic biomarkers of the antioxidant defense system were simultaneous analyzed on three occasions by the same laboratory using the same measurement methods and equipment. The data collection followed standardized protocols by trained staff. We controlled for several external and endogenous factors that could influence the biomarker status by defining appropriate exclusion criteria, by performing sensitivity analyses and by adjusting for these factors within analysis.

## 5. Conclusions

In conclusion, the majority of the assessed biomarkers of the antioxidant defense system exhibit a fair to very good reliability in healthy premenopausal women, but also a high degree of individuality. Thus, the RCV is more appropriate than population-based reference values to scrutinize and classify intra-individual changes in these biomarkers. Lipid soluble biomarkers, such as plasma α-carotene, plasma β-carotene and coenzyme Q10 as well as eGPx are the most reliable biomarkers, whereas glutathione and TEAC exhibit the poorest reliability owing to their very low inter-individual variance in this study population. To scrutinize the antioxidant defense system in more depth, the use of multiple measurements and biomarkers is advisable.

## Figures and Tables

**Table 1:** Descriptive characteristics of the subjects at each measurement time point (*n* = 44) ^a^.

Variable	Baseline	First Follow-Up	Second Follow-Up
**Biomarkers of antioxidant status**			
Serum bilirubin (µmol/L)	7.27 (5.13, 10.3) ^†^	6.84 (5.13, 9.83) ^†^	5.99 (3.42, 8.55) ^†^
Serum uric acid (µmol/L)	238 (214, 275) ^†^	242 (214, 289) ^†^	242 (208, 272) ^†^
Plasma vitamin C (µmol/L)	94 (81.8, 105) ^†^	91.1 (80.6, 100) ^†^	97.1 (85.5, 101) ^†^
Plasma α-tocopherol (µmol/L)	20.4 (17.5, 23.6) ^†^	18.4 (16.8, 20.4) ^‡^	17.7 (16.1, 20.5) ^‖^
Plasma γ-tocopherol (µmol/L)	1.03 (0.79, 1.38) ^†^	0.93 (0.69, 1.30) ^†^	0.90 (0.60, 1.24) ^†^
Plasma retinol (µmol/L)	2.04 (1.66, 2.31) ^†^	1.83 (1.49, 2.05) ^‡^	1.56 (1.27, 1.76) ^‖^
Plasma α-carotene (µmol/L)	0.34 (0.22, 0.48) ^†^	0.30 (0.22, 0.40) ^†^	0.27 (0.17, 0.40) ^†^
Plasma β-carotene (µmol/L)	0.89 (0.54, 1.34) ^†^	0.91 (0.59, 1.20) ^†^	0.86 (0.52, 1.24) ^†^
Plasma lutein (µmol/L)	0.21 (0.16, 0.29) ^†^	0.25 (0.18, 0.36) ^‡^	0.28 (0.21, 0.34) ^‡^
Plasma zeaxanthin (µmol/L)	0.11 (0.08, 0.16) ^†^	0.12 (0.08, 0.16) ^†,‡^	0.14 (0.09, 0.18) ^‡^
Plasma β-cryptoxanthin (µmol/L)	0.22 (0.16, 0.31) ^†^	0.21 (0.14, 0.27) ^‡^	0.17 (0.13, 0.24) ^‖^
Plasma lycopene (µmol/L)	0.74 (0.59, 0.99) ^†^	0.72 (0.57, 0.92) ^†^	0.72 (0.59, 0.95) ^†^
Serum coenzyme Q10 (nmol/L)	515 (426, 650) ^†^	601 (474, 772) ^‡^	569 (411, 734) ^†,‡^
Whole blood glutathione (µmol/L)	656 (572, 765) ^†^	736 (675, 792) ^‡^	667 (618, 731) ^†^
eCAT (kU/gHb) ^b^	638 (589, 687) ^†^	662 (594, 710) ^†^	648 (602, 686) ^†^
eGPx (U/gHb) ^b^	24.5 (20.9, 27.7) ^†^	27.4 (22.1, 31.5) ^‡^	25.5 (21.9, 30.4) ^‡^
Plasma TEAC (mmol/L)	1.11 (1.05, 1.17) ^†^	1.18 (1.14, 1.22) ^‡^	1.24 (1.20, 1.28) ^‖^
**Blood count and hs-CRP**			
Hemoglobin (g/dL)	12.8 (12.1, 13.4)	12.4 (11.8, 13.1)	12.4 (11.6, 12.8)
Hematocrit (%)	38.3 (36.4, 39.4)	37.0 (35.6, 38.6)	36.9 (35.2, 38.0)
Red blood cells (×10⁶/µL)	4.34 (4.13, 4.56)	4.25 (4.03, 4.43)	4.17 (3.99, 4.42)
White blood cells (×10^3^/µL)	4.65 (3.65, 5.67)	4.53 (3.88, 5.66)	4.45 (3.82, 5.21)
Platelets (×10^3^/µL)	239 (201, 292)	226 (198, 276)	231 (204, 272)
Serum hs-CRP (nmol/L)	10.2 (4.19, 20.1)	7.29 (3.33, 20.0)	7.57 (3.14, 20.4)
**Serum lipids**			
Serum cholesterol (mmol/L)	3.85 (3.27, 4.68)	3.94 (3.51, 4.40)	3.80 (3.47, 4.38)
Serum triglycerides (mmol/L)	0.80 (0.59, 1.03)	0.74 (0.60, 0.94)	0.70 (0.57, 1.07)
**Data on diet**			
Vegan/Vegetarian diet (%)	41	39	39
Energy (kJ/d) ^b^	8113 (6709, 9599)	8031 (6703, 9161)	8159 (7324, 9388)
Alcohol (g/d) ^b^	0.10 (0.0, 1.25)	0.90 (0.0, 7.45)	0.10 (0.0, 5.80)
Vitamin C (mg/d) ^b^	147 (106, 225)	129 (79.6, 177)	141 (95.9, 201)
Tocopherol equivalents (mg/d) ^b^	14.0 (10.5, 19.6)	13.3 (11.2, 18.2)	12.1 (9.80, 16.0)
Retinol equivalents (mg/d) ^b^	1.45 (1.10, 2.42)	1.20 (0.81, 2.14)	1.28 (0.93, 2.01)
β-Carotene (mg/d) ^b^	6.05 (4.05, 10.8)	4.25 (2.25, 8.95)	4.70 (1.90, 9.70)
Purine (mg/d) ^b^	120 (96.1, 146)	114 (89.7, 155)	122 (103, 145)
Time since last meal (h)	13.3 (12.8, 13.9)	13.3 (12.7, 14.3)	12.9 (12.6, 13.3)
**Data on BMI and lifestyle**			
BMI (kg/m^2^)	21.8 (20.5, 24.4)	21.6 (20.3, 24.5)	21.7 (20.4, 24.4)
Sleep duration, last night (h)	8.0 (7.2, 8.7)	8.0 (7.6, 8.6)	7.9 (7.2, 8.7)
Physical activity level	1.59 (1.47, 1.68)	1.57 (1.50, 1.70)	1.60 (1.52, 1.69)
**Data on hormonal status**			
Time since last menstrual period (d) ^c^	15.0 (7.0, 22.0)	18.5 (10.0, 23.5)	17.0 (8.0, 23.0)
Use of contraceptives (%)	50	50	48

Abbreviations: BMI, body mass index; eCAT, erythrocyte catalase; Hb, hemoglobin; eGPx, erythrocyte glutathione peroxidase; TEAC, Trolox equivalent antioxidant capacity. ^a^ Data are presented as median and interquartile range for continuous variables and absolute and relative frequencies for categorical variables. Differences in antioxidant biomarkers across measurement time points were tested using the Friedman test and Wilcoxon signed-rank tests with Bonferroni adjustment were performed if the overall test was significant. Different superscript symbols for values in the same row indicate significant differences between time points. ^b^ Missing data were noted for eCAT (*n* = 4), eGPx (*n* = 4) and dietary record (*n* = 1) at least in one assessment, thus sample size was reduced to 40 subjects for eCAT/eGPx analysis and to 43 subjects for data analysis of dietary records, respectively. ^c^ Refers to the median time elapsed since the first day of the last menstrual period.

**Table 2:** Crude and adjusted indicators of reliability and inter- and intra-individual variability for each antioxidant biomarker in premenopausal women ^a,b^.

Biomarkers of Antioxidant Status	*n* ^c^	ICC_unadj_ [95% CI]	CV_G_	CV_T_	II	RCV_%,pos_	RCV_%,neg_	n_β,10%_	n_β,20%_	ICC_adj_ [95% CI] ^d^
Serum bilirubin (µmol/L)	42	0.567 [0.380, 0.710]	0.343	0.299	0.874	±83.0		7	3	0.558 [0.393, 0.737]
Serum uric acid (µmol/L) ^e^	44	0.693 [0.540, 0.801]	0.170	0.113	0.663	+36.6	−26.8	4	2	0.682 [0.535, 0.810]
Plasma vitamin C (µmol/L) ^e^	44	0.515 [0.326, 0.668]	0.127	0.123	0.970	+40.6	−28.9	9	4	0.529 [0.344, 0.704]
Plasma α-tocopherol (µmol/L) ^e^	44	0.700 [0.548, 0.806]	0.169	0.110	0.652	+35.6	−26.2	4	2	0.541 [0.360, 0.711]
Plasma γ-tocopherol (nmol/L) ^e^	43	0.628 [0.442, 0.753]	0.409	0.310	0.757	+131.6	−56.8	6	3	0.549 [0.377, 0.724]
Plasma retinol (nmol/L) ^e^	44	0.514 [0.325, 0.667]	0.191	0.186	0.972	+66.7	−40.0	9	4	0.435 [0.239, 0.633]
Plasma α-carotene (nmol/L) ^e^	40	0.793 [0.673, 0.869]	0.547	0.266	0.487	+106.4	−51.5	3	1	0.783 [0.676, 0.885]
Plasma β-carotene (nmol/L) ^e^	41	0.875 [0.796, 0.924]	0.617	0.217	0.352	+81.2	−44.8	2	1	0.832 [0.747, 0.906]
Plasma lutein (nmol/L) ^e^	43	0.681 [0.509, 0.792]	0.336	0.227	0.675	+86.1	−46.3	5	2	0.414 [0.224, 0.627]
Plasma zeaxanthin (nmol/L) ^e^	44	0.514 [0.325, 0.668]	0.333	0.323	0.316	+139.7	−58.3	9	4	0.421 [0.222, 0.621]
Plasma β-cryptoxanthin (nmol/L) ^e^	41	0.758 [0.626, 0.846]	0.378	0.209	0.552	+77.2	−43.6	3	2	0.781 [0.669, 0.877]
Plasma lycopene (nmol/L) ^e^	44	0.699 [0.547, 0.805]	0.385	0.248	0.644	+96.9	−49.2	4	2	0.447 [0.251, 0.643]
Serum coenzyme Q10 (nmol/L) ^e^	44	0.760 [0.627, 0.846]	0.300	0.166	0.554	+58.1	−36.7	3	2	0.578 [0.405, 0.739]
eCAT (kU/gHb)	40	0.658 [0.493, 0.776]	0.083	0.060	0.721	±16.7		5	2	0.602 [0.432, 0.771]
eGPx (U/gHb) ^e^	40	0.765 [0.634, 0.850]	0.236	0.130	0.549	+43.1	−30.1	3	2	0.801 [0.698, 0.892]
Blood glutathione (µmol/L)	43	0.098 [0.000, 0.286]	0.053	0.161	3.030	±44.6		83	37	0.072 [0.000, 0.313]
Plasma TEAC (mmol/L) ^f^	43	0.000 [0.000, 0.175]	0.000	0.070	/	±19.3		/	/	0.000 [0.000, 0.235]

Abbreviations: ICC, intraclass correlation coefficient; CV_G_, inter-individual variation; CV_T_, total intra-individual variation; II, index of individuality; RCV, reference change value; n_β,10%_, number of required measurements to limit the attenuation in regression coefficient to 10%; n_β,20%_, number of required measurements to limit the attenuation in regression coefficient to 20%; eCAT, erythrocyte catalase; Hb, hemoglobin; eGPx, erythrocyte glutathione peroxidase; TEAC, Trolox equivalent antioxidant capacity. ^a^ For each considered subject, three mean values of the respective blood biomarker were available. ^b^ Except for the adjusted ICC, variance components were estimated by restricted maximum likelihood method via one-way random effect model. ^c^ Sample size after excluding pre-identified outliers by Cochran’s test and Reed’s criterion. The original sample size was 44 subjects for all biomarkers except for eCAT and eGPx, for which the original sample size was 40 subjects. ^d^ Adjusted ICC was calculated using the variance components estimated by restricted maximum likelihood method via a linear mixed-effects model using the following fixed effects: time elapsed since the first day of the last menstrual period (d), body mass index (kg/m^2^), fasting duration (h), sleep duration of the last night (h), use of contraceptives (no vs. yes), serum cholesterol concentration (mmol/L) and physical activity level. In addition, energy intake (MJ/d) or, if applicable, nutrient intake levels (i.e., purine intake (mg/d) for serum uric acid, vitamin C intake (mg/d) for plasma vitamin C, tocopherol equivalents (mg/d) for both plasma tocopherol parameters, retinol equivalents (mg/d) for plasma retinol and β-carotene (mg/d) for plasma carotenoids) were included in the model, respectively. For each of these fixed effects, data from all three measuring time points were considered. Sample size was reduced by one subject because one subject provided no dietary record in the third assessment. ^e^ Biomarker values were logarithmically transformed (natural logarithm). ^f^ Model with a singular fit.

**Table 3:** Indicators of reliability and inter- and intra-individual variability for each antioxidant biomarker in premenopausal women after excluding subjects with conditions that may impair or influence biomarker status ^a^.

Biomarkers of Antioxidant Status	*n* ^b^	ICC_unadj_ [95% CI]	CV_G_	CV_T_	II	RCV_%,pos_	RCV_%,neg_	n_β,10%_	n_β,20%_
Serum bilirubin (µmol/L)	18	0.605 [0.302, 0.776]	0.383	0.309	0.808	±85.7		6	3
Serum uric acid (µmol/L) ^c^	17	0.506 [0.143, 0.724]	0.126	0.124	0.989	+41.0	−29.1	9	4
Plasma vitamin C (µmol/L) ^c^	16	0.615 [0.257, 0.808]	0.122	0.097	0.790	+30.7	−23.5	6	3
Plasma α-tocopherol (µmol/L) ^c^	16	0.771 [0.504, 0.893]	0.146	0.079	0.543	+24.6	−19.7	3	1
Plasma γ-tocopherol (nmol/L) ^c^	14	0.710 [0.383, 0.862]	0.330	0.208	0.630	+76.8	−43.4	4	2
Plasma retinol (nmol/L) ^c^	17	0.575 [0.234, 0.770]	0.207	0.177	0.857	+62.9	−38.6	7	3
Plasma α-carotene (nmol/L) ^c^	12	0.880 [0.673, 0.950]	0.551	0.192	0.348	+69.5	−41.0	2	1
Plasma β-carotene (nmol/L) ^c^	13	0.977 [0.927, 0.990]	0.733	0.101	0.138	+32.2	−24.4	1	1 ^d^
Plasma lutein (nmol/L) ^c^	16	0.731 [0.433, 0.872]	0.344	0.205	0.596	+75.3	−43.0	4	2
Plasma zeaxanthin (nmol/L) ^c^	13	0.883 [0.679, 0.949]	0.461	0.161	0.349	+55.8	−35.8	2	1
Plasma β-cryptoxanthin (nmol/L) ^c^	16	0.610 [0.252, 0.805]	0.202	0.161	0.797	+55.8	−35.8	6	3
Plasma lycopene (nmol/L) ^c^	18	0.797 [0.585, 0.895]	0.467	0.227	0.486	+86.1	−46.3	3	1
Serum coenzyme Q10 (nmol/L) ^c^	17	0.758 [0.500, 0.880]	0.286	0.160	0.558	+55.3	−35.6	3	2
eCAT (kU/gHb)	14	0.702 [0.368, 0.858]	0.095	0.062	0.652	±17.2		4	2
eGPx (U/gHb) ^c^	13	0.829 [0.564, 0.923]	0.219	0.099	0.451	+31.4	−23.9	2	1
Blood glutathione (µmol/L)	14	0.477 [0.085, 0.724]	0.094	0.099	1.047	±27.4		10	5
Plasma TEAC (mmol/L) ^e^	17	0.000 [0.000, 0.300]	0.000	0.078	/	±21.7		/	/

Abbreviations: ICC, intraclass correlation coefficient; CV_G_, inter-individual variation; CV_T_, total intra-individual variation; II, index of individuality; RCV, reference change value; n_β,10%_, number of required measurements to limit the attenuation in regression coefficient to 10%; n_β,20%_, number of required measurements to limit the attenuation in regression coefficient to 20%; eCAT, erythrocyte catalase; Hb, hemoglobin; eGPx, erythrocyte glutathione peroxidase; TEAC, Trolox equivalent antioxidant capacity. ^a^ For each considered subject, three mean values of the respective blood biomarker were available. Variance components were estimated by restricted maximum likelihood method via one-way random effect model. ^b^ Sample size after excluding pre-identified outliers by Cochran’s test and Reed’s criterion as well as subjects with at least one model-wise outlying residuum (i.e., >2.0) of the applied model. The sample size before exclusion of outliers was 18 subjects for all biomarkers except for eCAT and eGPx, for which the initial sample size was 15 subjects. ^c^ Biomarker values were logarithmically transformed (natural logarithm). ^d^ Calculation resulted in n_β,20%_ below 0.2. ^e^ Model with a singular fit.

## Data Availability

The anonymized datasets of this study are available from the corresponding author on reasonable request.

## Supplementary Materials

The following are available online at https://www.mdpi.com/2076-3921/10/3/448/s1, Figure S1: Flow chart of the present investigation. Figure S2: Correlation matrix based on Spearman correlation analyses at baseline. Figure S3: Mean values and absolute ranges of antioxidant biomarkers for each subject over the study period. Table S1: Crude and adjusted indicators of reliability and inter- and intra-individual variability for each antioxidant biomarker in premenopausal women after excluding subjects with outlying residuals.

## Abbreviations

95% CI	95% confidence interval
BMI	body mass index
CV_G_	inter-individual coefficient of variation
CV_T_	total intra-individual coefficient of variation
eCAT	erythrocyte catalase
EDTA	ethylenediaminetetraacetic acid
eGPx	erythrocyte glutathione peroxidase
hs-CRP	high-sensitive C-reactive protein
ICC	intraclass correlation coefficient
II	index of individuality
MET	metabolic equivalents
n_β_	number of required measurements to limit the attenuation in regression coefficient
PAL	physical activity level
RCV	reference change value
RMR	resting metabolic rate
TEAC	Trolox equivalent antioxidant capacity
