# Supplementary material for: Intra-Individual Variation and Reliability of Biomarkers of the Antioxidant Defense System by Considering Dietary and Lifestyle Factors in Premenopausal Women
Source: Antioxidants (Basel). 2021 Mar 13;10(3):448. doi: 10.3390/antiox10030448 (PMC7998493; doi:10.3390/antiox10030448)
Supplement: Supplementary file 1 [file antioxidants-10-00448-s001.pdf]

Intra-individual variation and reliability of biomarkers of the antioxidant defense system by considering dietary and lifestyle factors in premenopausal women

Supplementary Material; Jungert, A.; Frank, J.

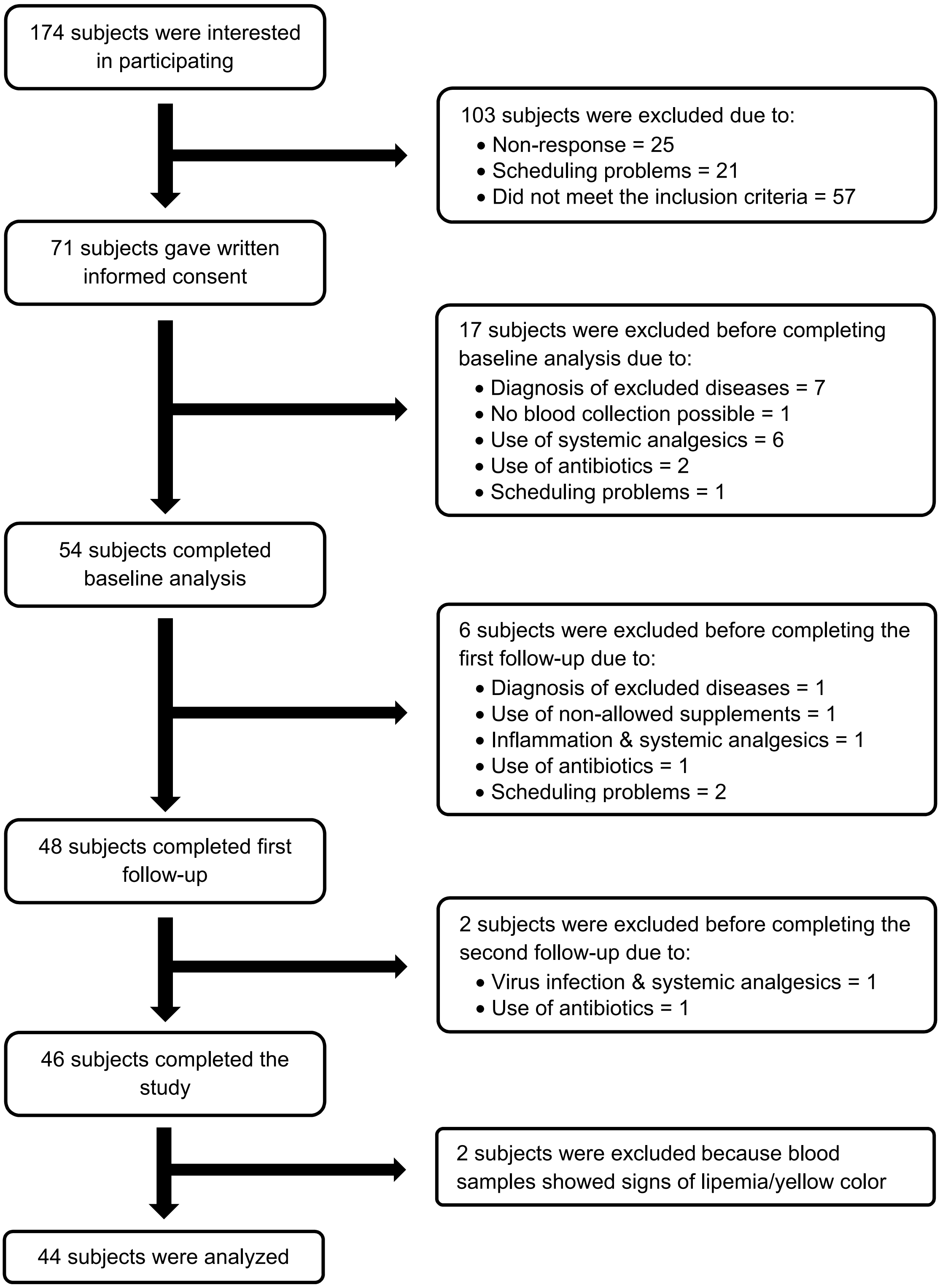

Supplementary Figure S1 Flow chart of the present investigation

This flow chart illustrates the numbers and reasons for excluding subjects throughout the study period. Successful completion means in this context that the subject completed the physical assessment as well as questionnaires and reported no exclusion criteria. In total, 174 subjects were interested in participating in the study and 46 subjects completed the study by participating in all three examinations, i.e., baseline, first follow-up and second follow-up. The final sample size was 44 subjects.

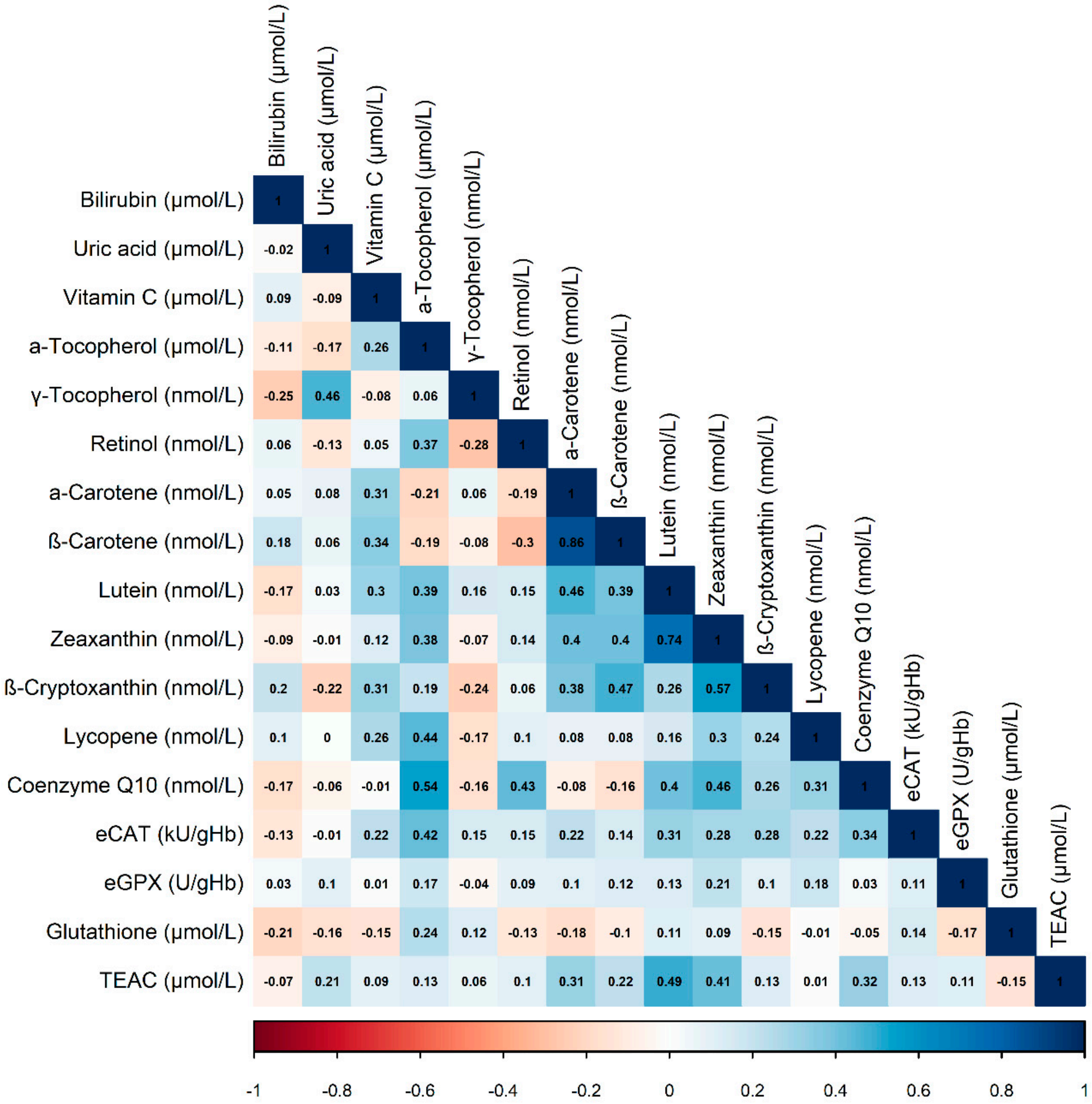

Supplementary Figure S2 Correlation matrix based on Spearman correlation analyses at baseline (*n* = 44)

In addition to the Spearman correlation coefficient, the saturation of the red (negative association) and blue (positive association) color denotes the strength of the respective correlation. Four subjects had missing data on eCAT and eGPx measurements resulting in a sample size of 40 subjects at baseline. Abbreviations: eCAT, erythrocyte catalase; eGPx, erythrocyte glutathione peroxidase; TEAC, Trolox equivalent antioxidant capacity.

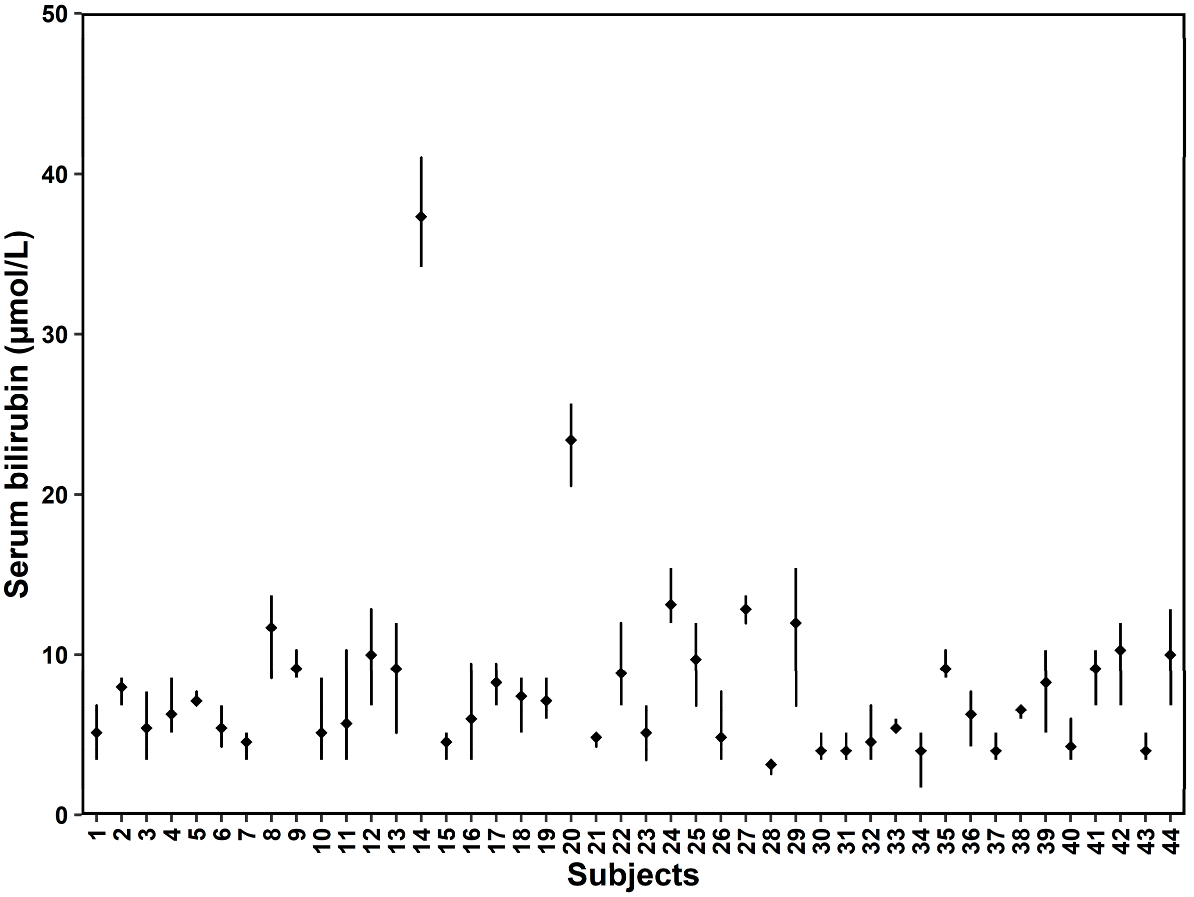

Serum bilirubin (µmol/L)
0
10
20
30
40
50
Subjects
1 2 3 4 5 6 7 8 9 10 11 12 13 14 15 16 17 18 19 20 21 22 23 24 25 26 27 28 29 30 31 32 33 34 35 36 37 38 39 40 41 42 43 44

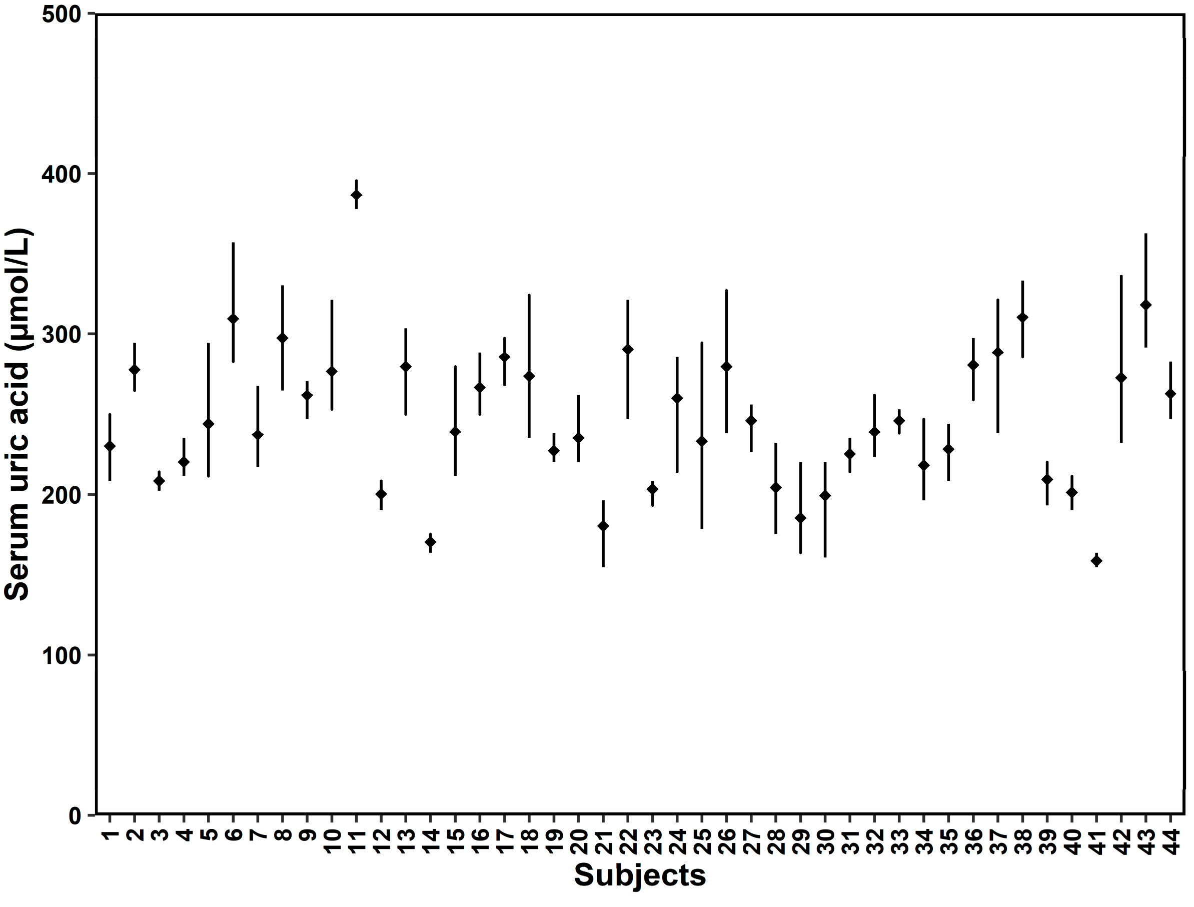

Serum uric acid (µmol/L)
0
100
200
300
400
500
Subjects
1 2 3 4 5 6 7 8 9 10 11 12 13 14 15 16 17 18 19 20 21 22 23 24 25 26 27 28 29 30 31 32 33 34 35 36 37 38 39 40 41 42 43 44

Intra-individual variation and reliability of biomarkers of the antioxidant defense system by considering dietary and lifestyle factors in premenopausal women

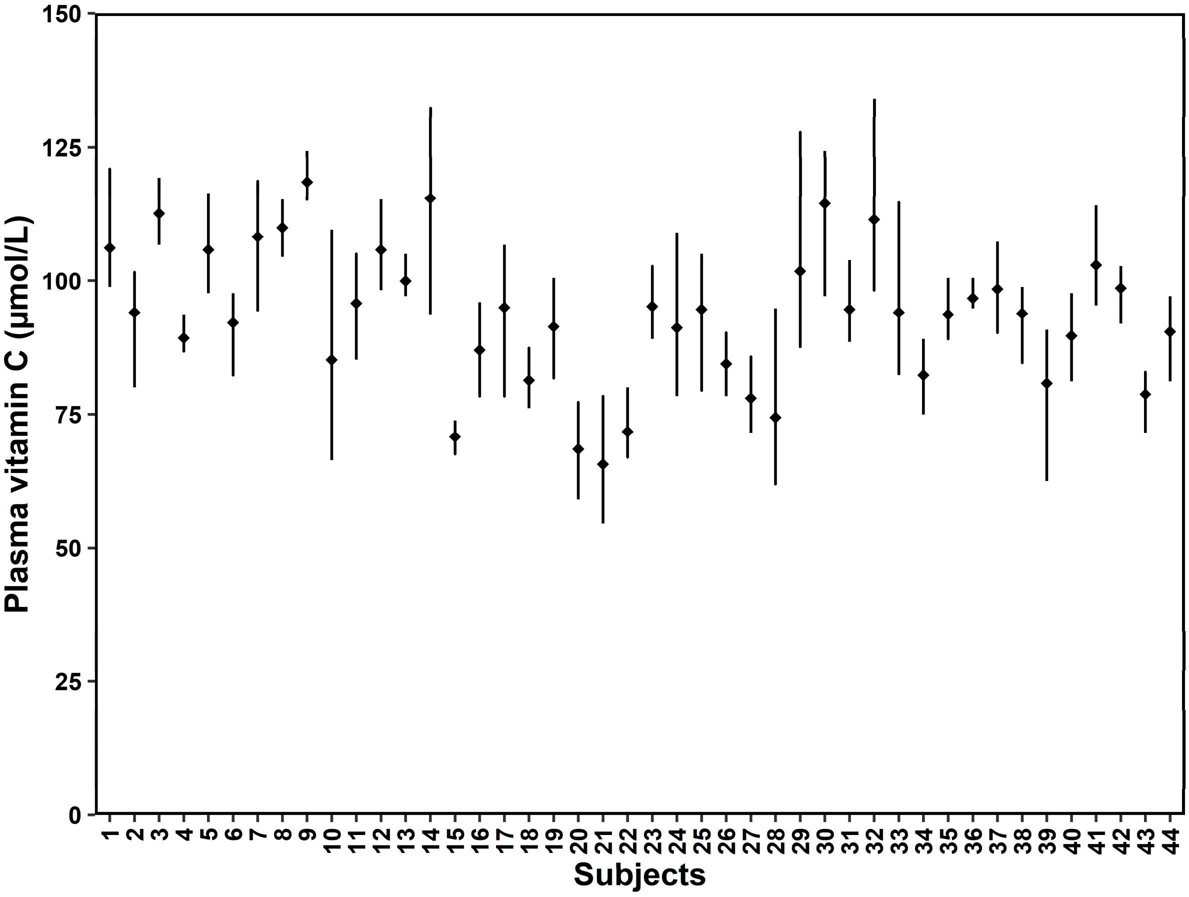

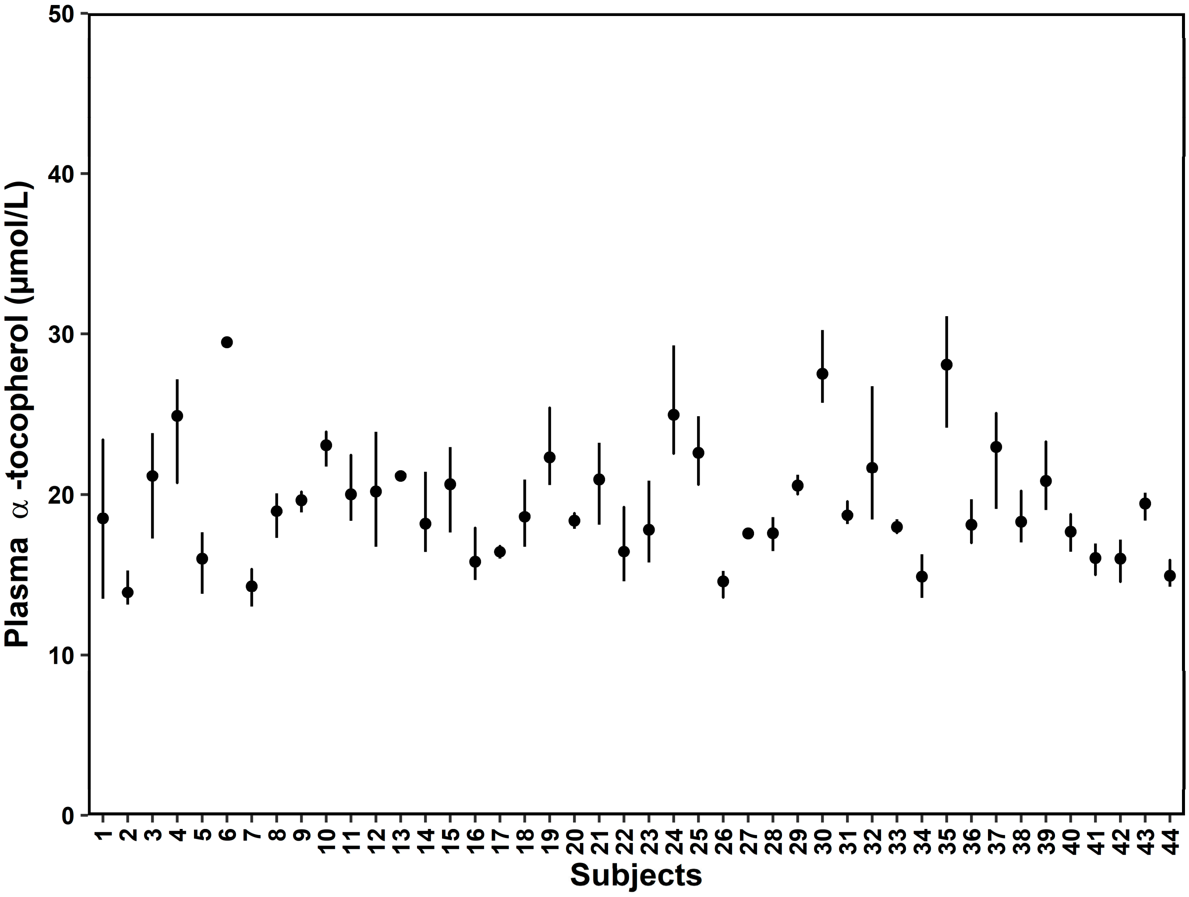

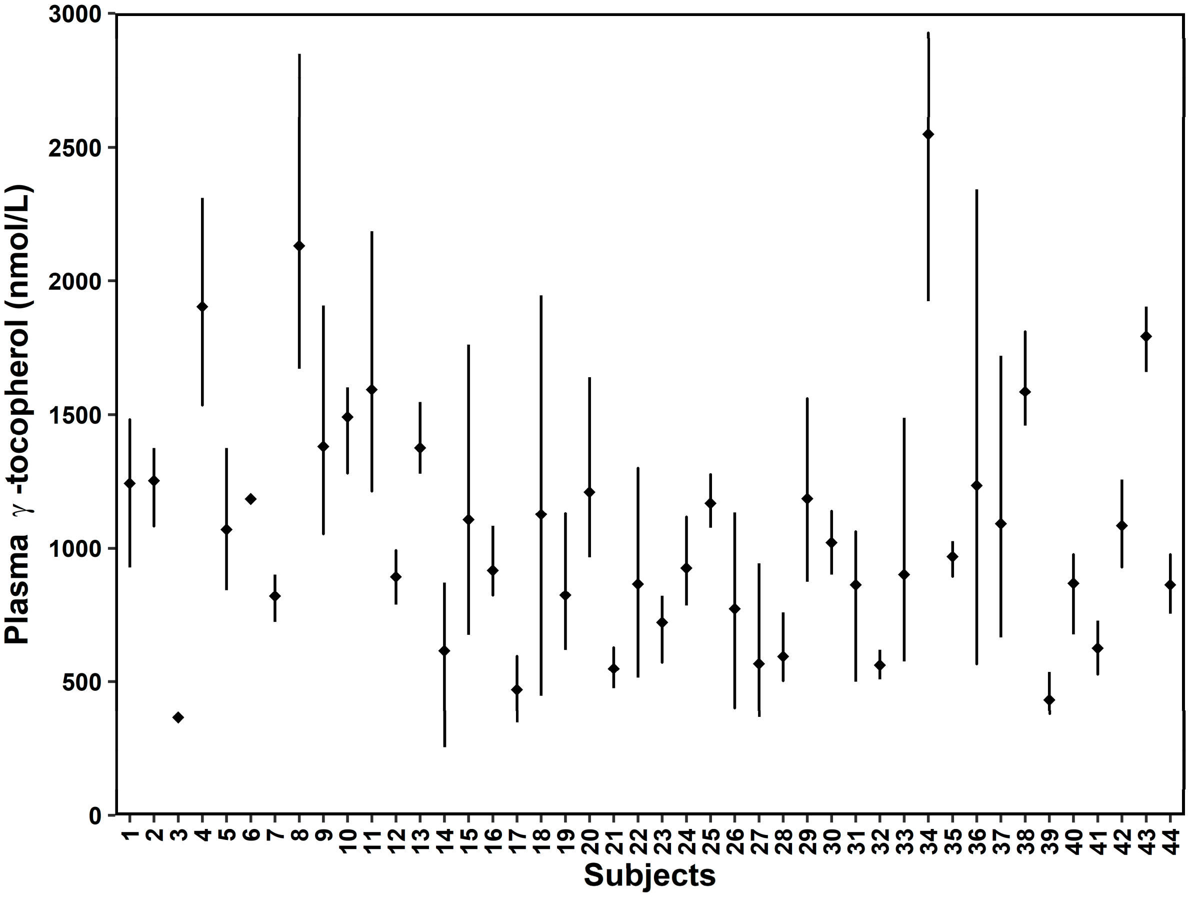

Plasma γ -tocopherol (nmol/L)
3000
2500
2000
1500
1000
500
0
1 2 3 4 5 6 7 8 9 10 11 12 13 14 15 16 17 18 19 20 21 22 23 24 25 26 27 28 29 30 31 32 33 34 35 36 37 38 39 40 41 42 43 44
Subjects

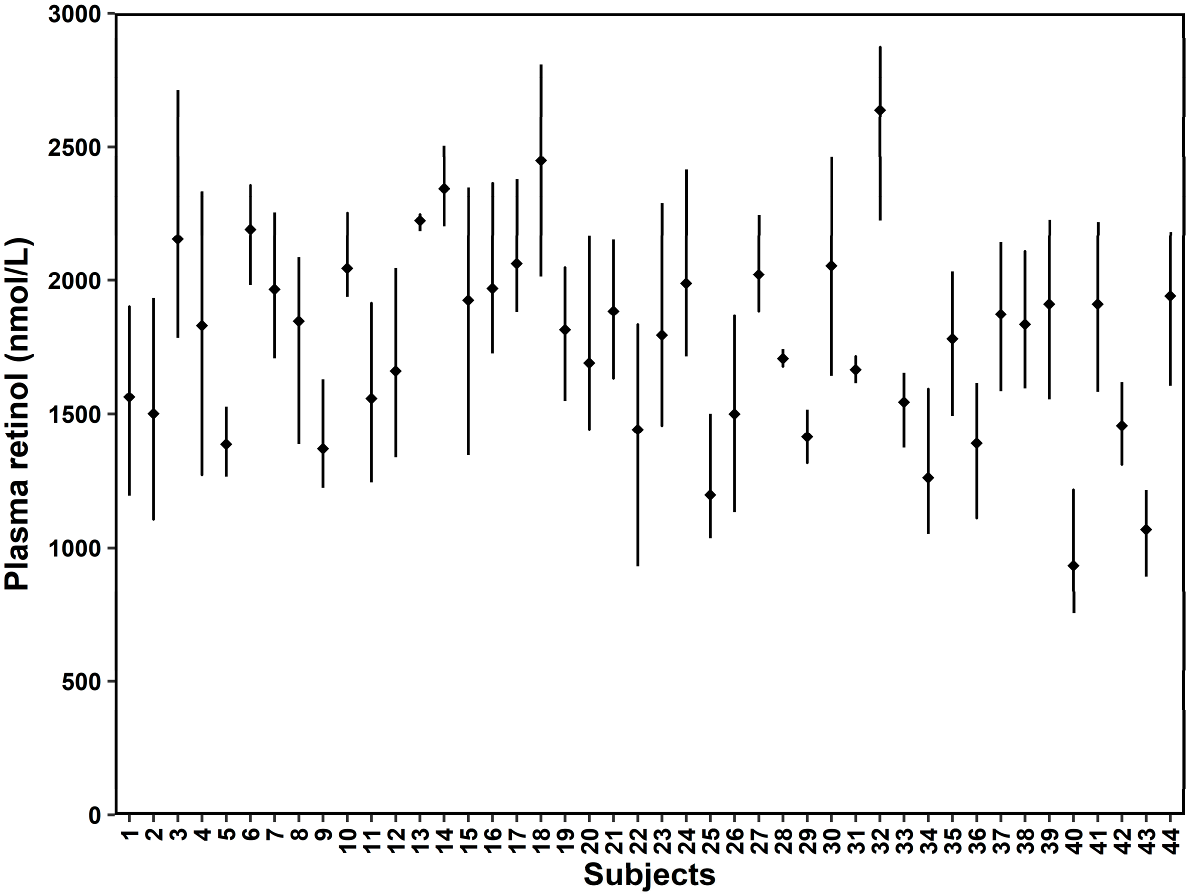

Plasma retinol (nmol/L)
3000
2500
2000
1500
1000
500
0
1 2 3 4 5 6 7 8 9 10 11 12 13 14 15 16 17 18 19 20 21 22 23 24 25 26 27 28 29 30 31 32 33 34 35 36 37 38 39 40 41 42 43 44
Subjects

Intra-individual variation and reliability of biomarkers of the antioxidant defense system by considering dietary and lifestyle factors in premenopausal women

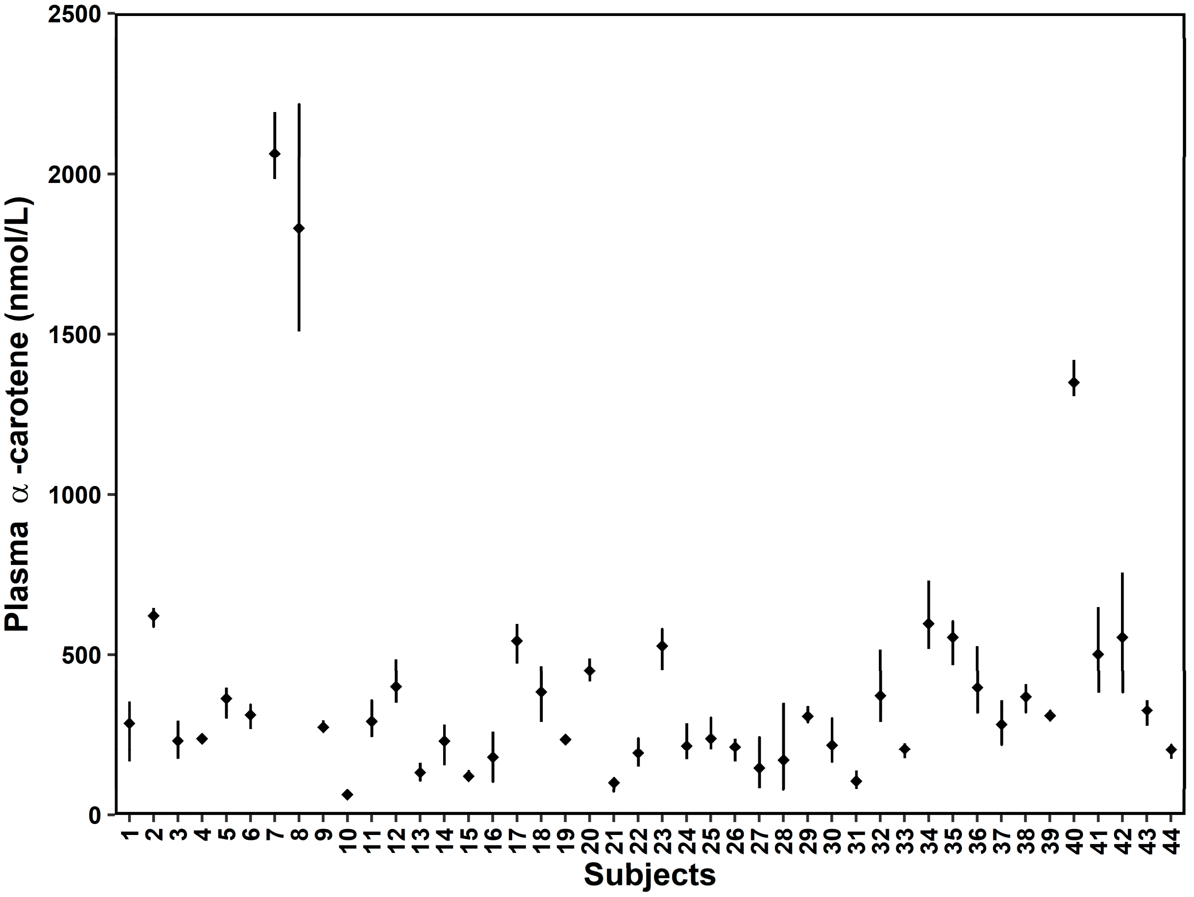

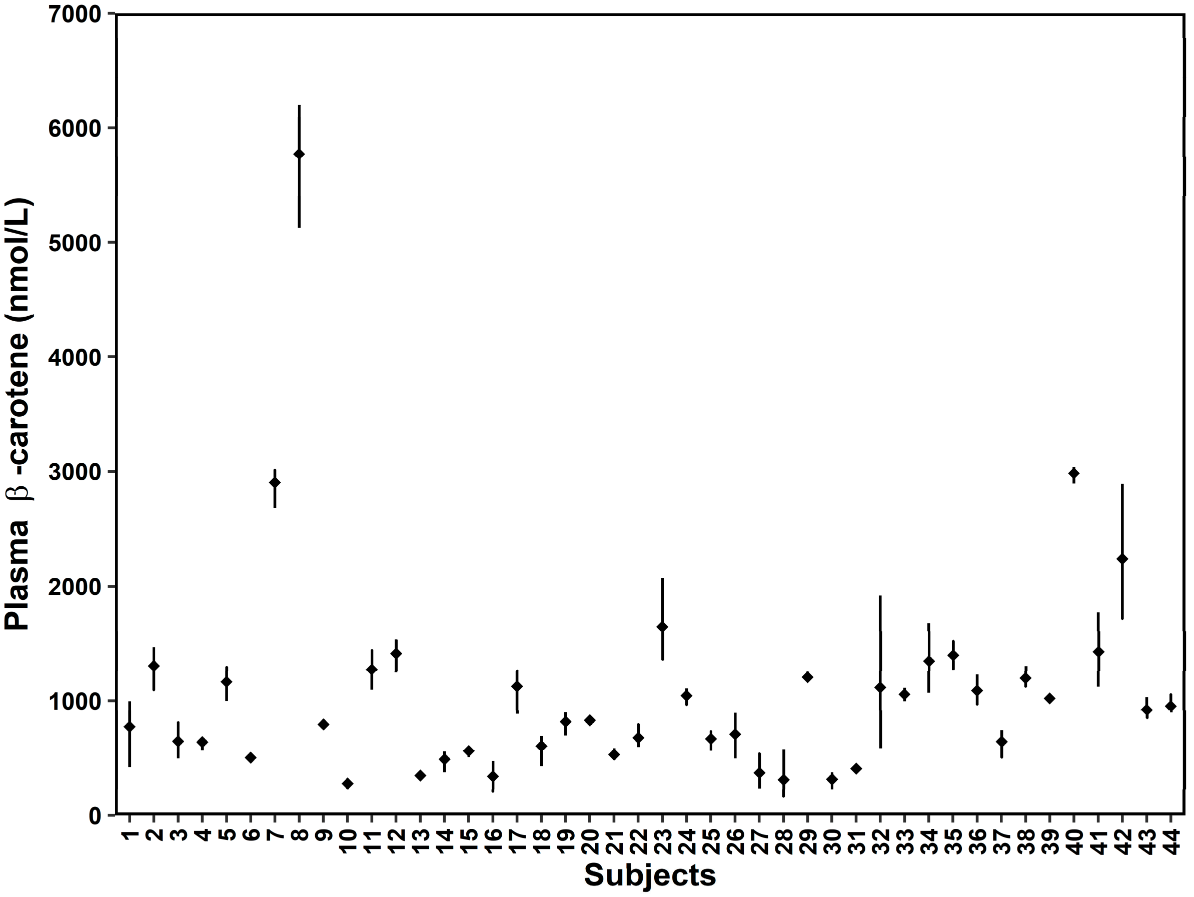

Intra-individual variation and reliability of biomarkers of the antioxidant defense system by considering dietary and lifestyle factors in premenopausal women

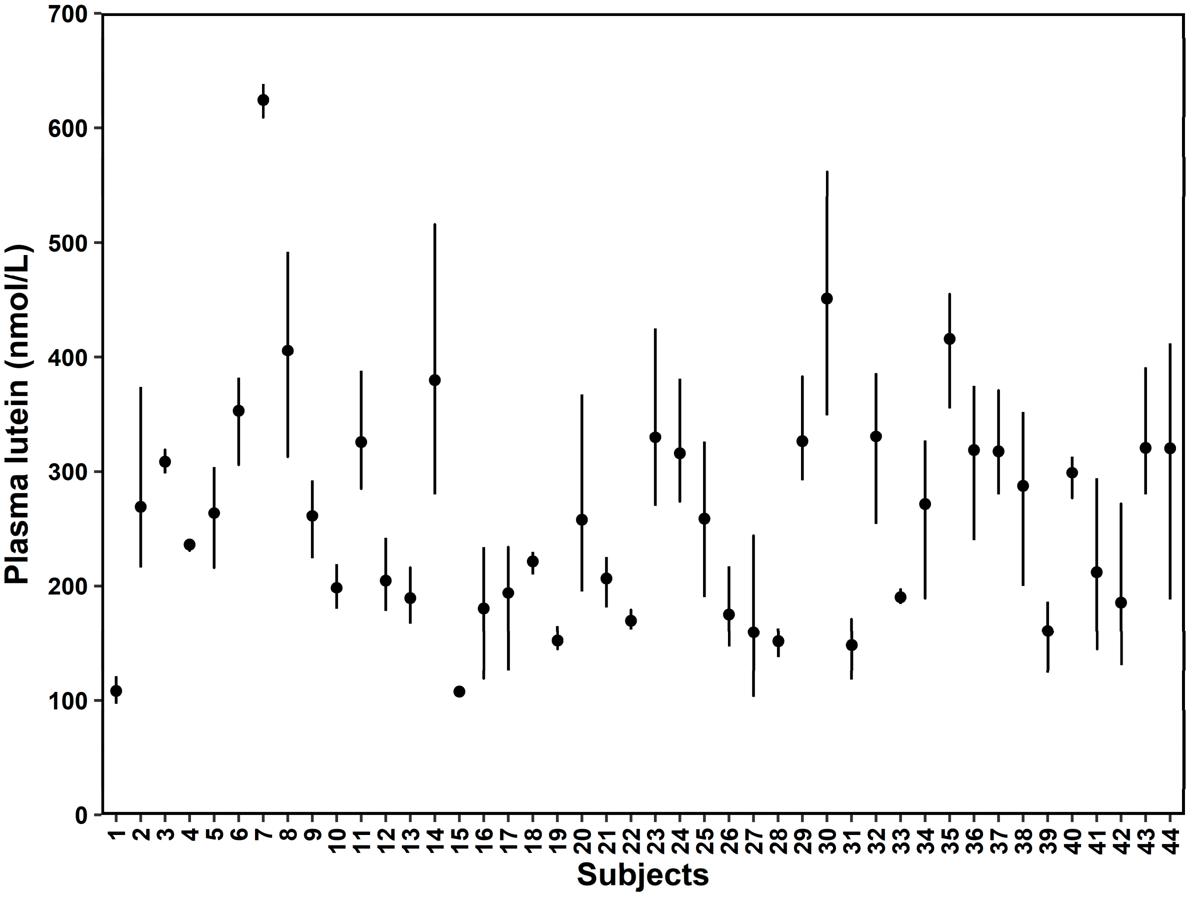

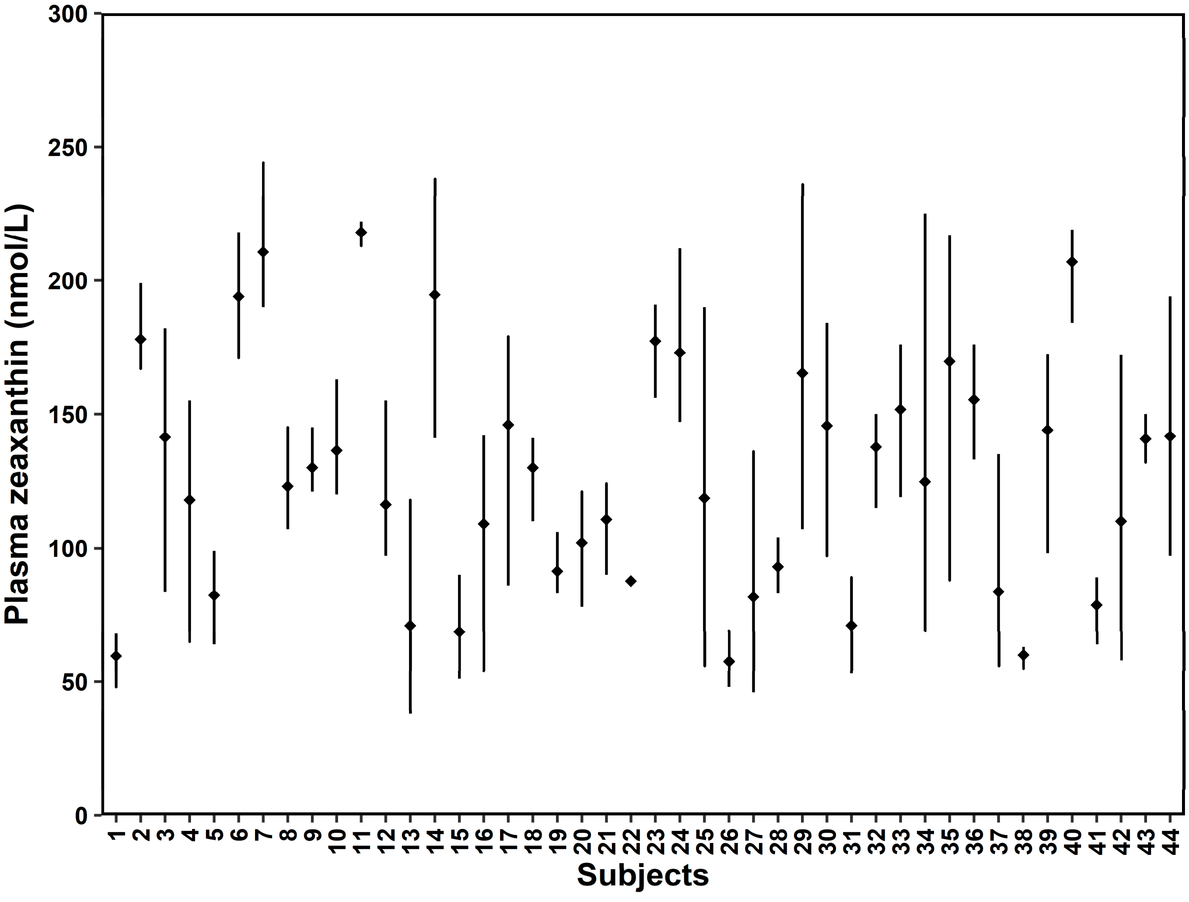

Intra-individual variation and reliability of biomarkers of the antioxidant defense system by considering dietary and lifestyle factors in premenopausal women

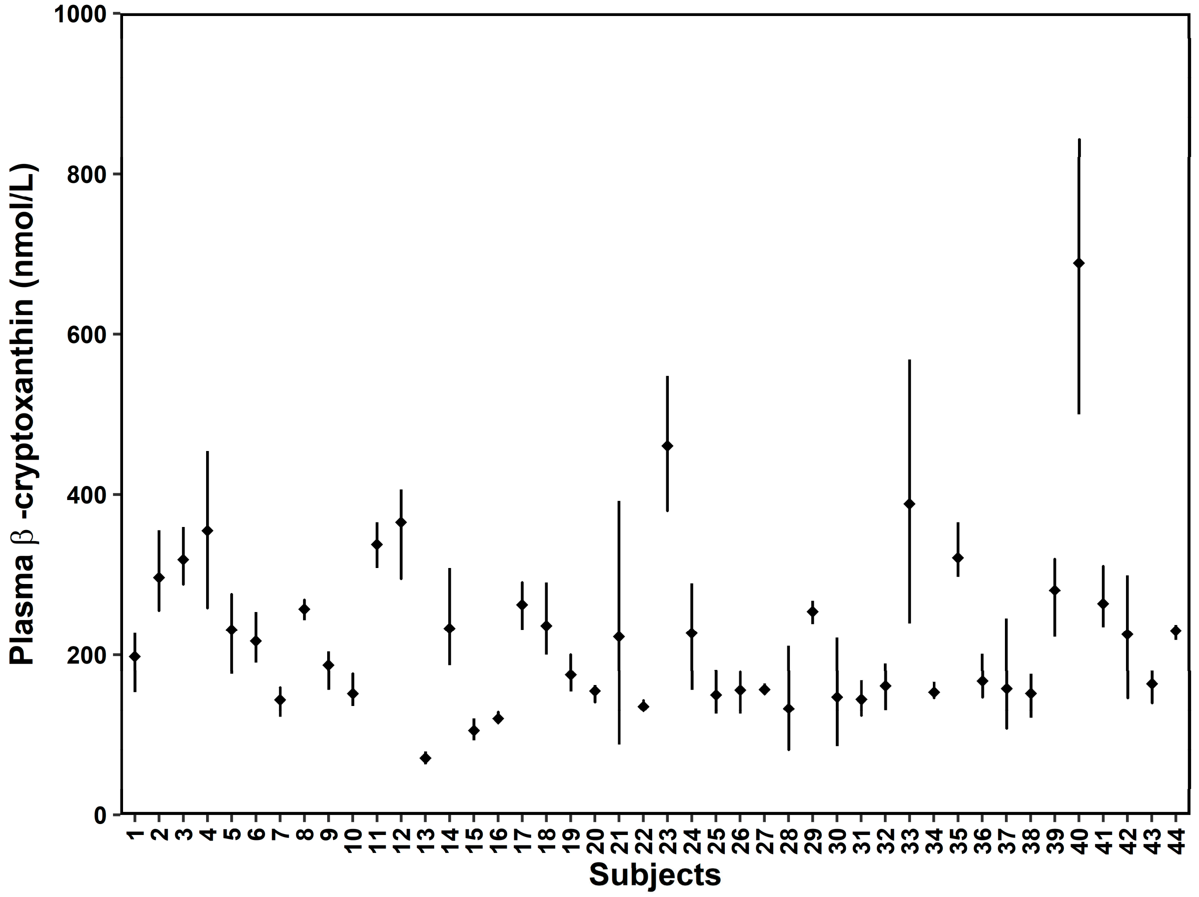

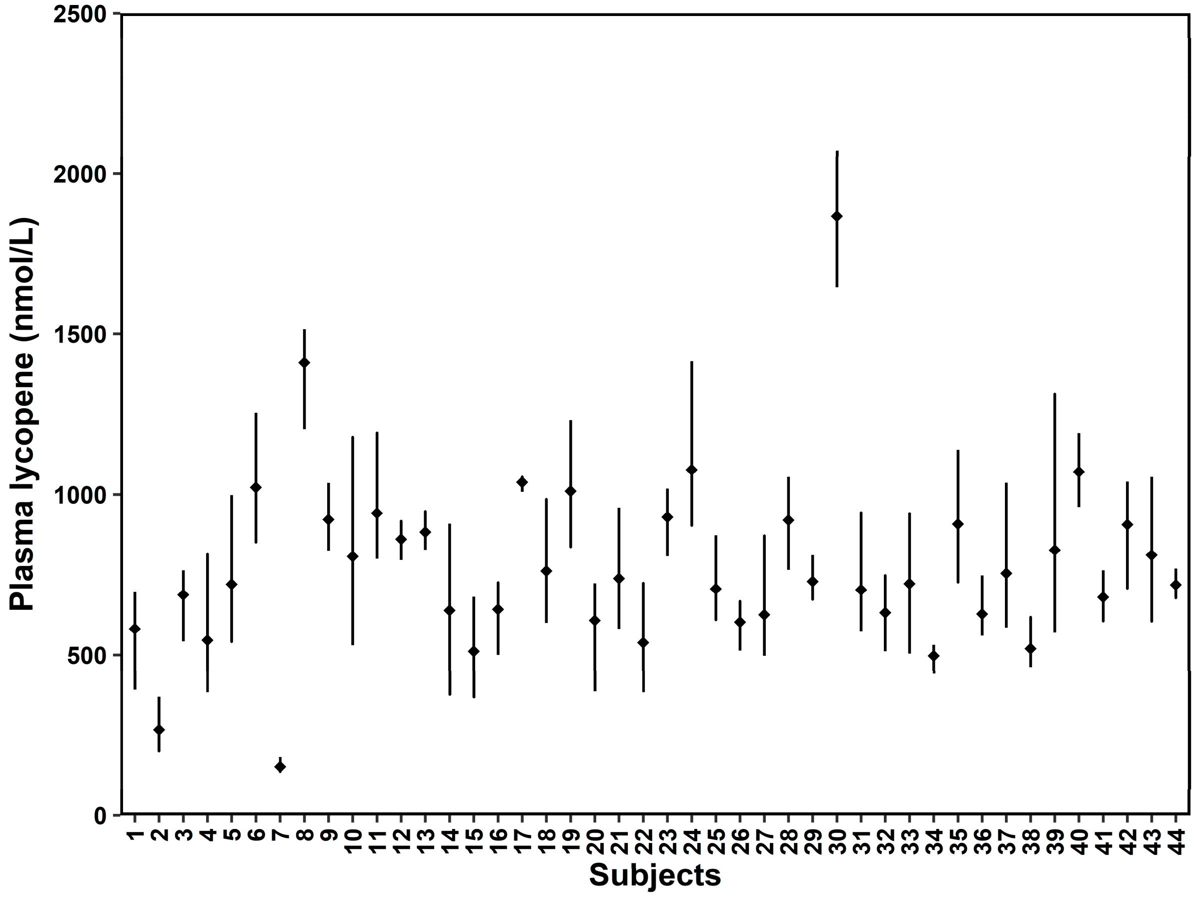

Intra-individual variation and reliability of biomarkers of the antioxidant defense system by considering dietary and lifestyle factors in premenopausal women

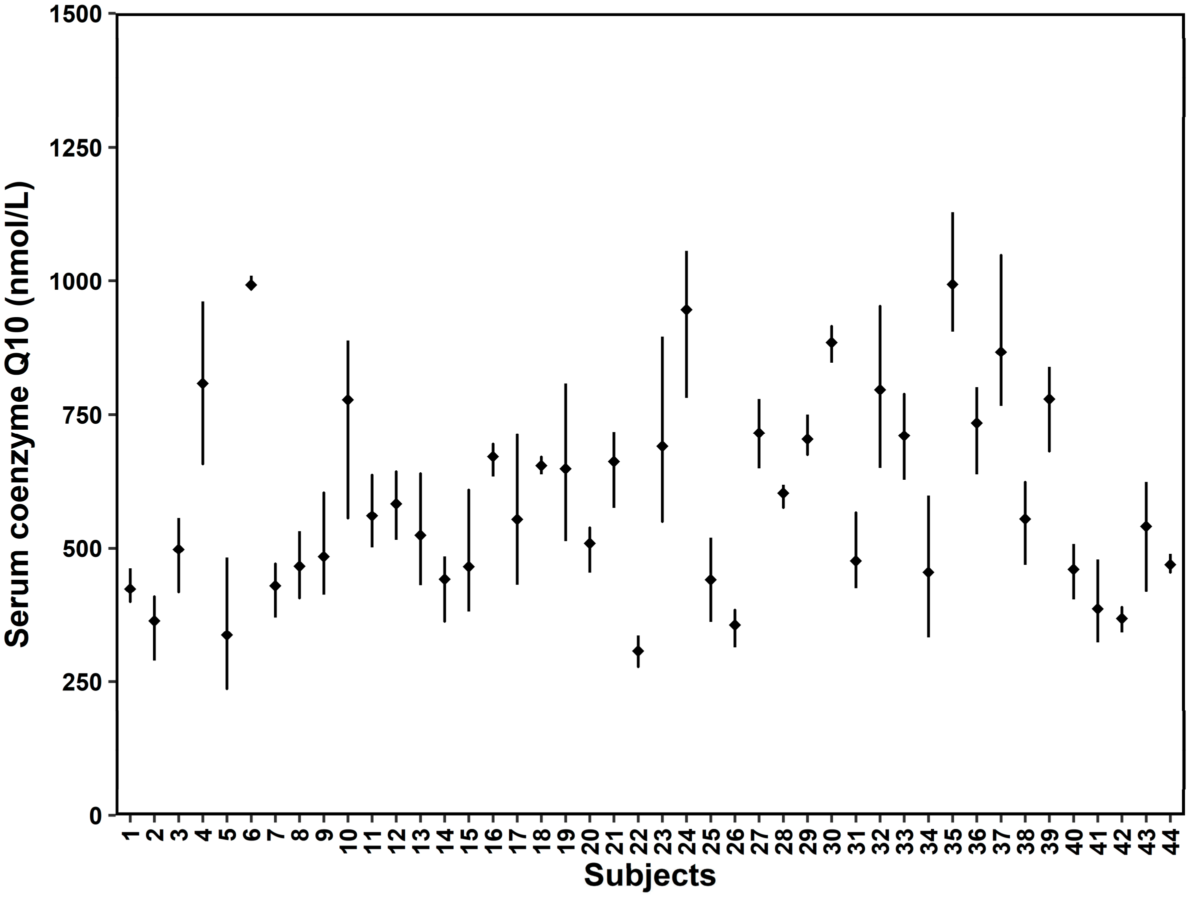

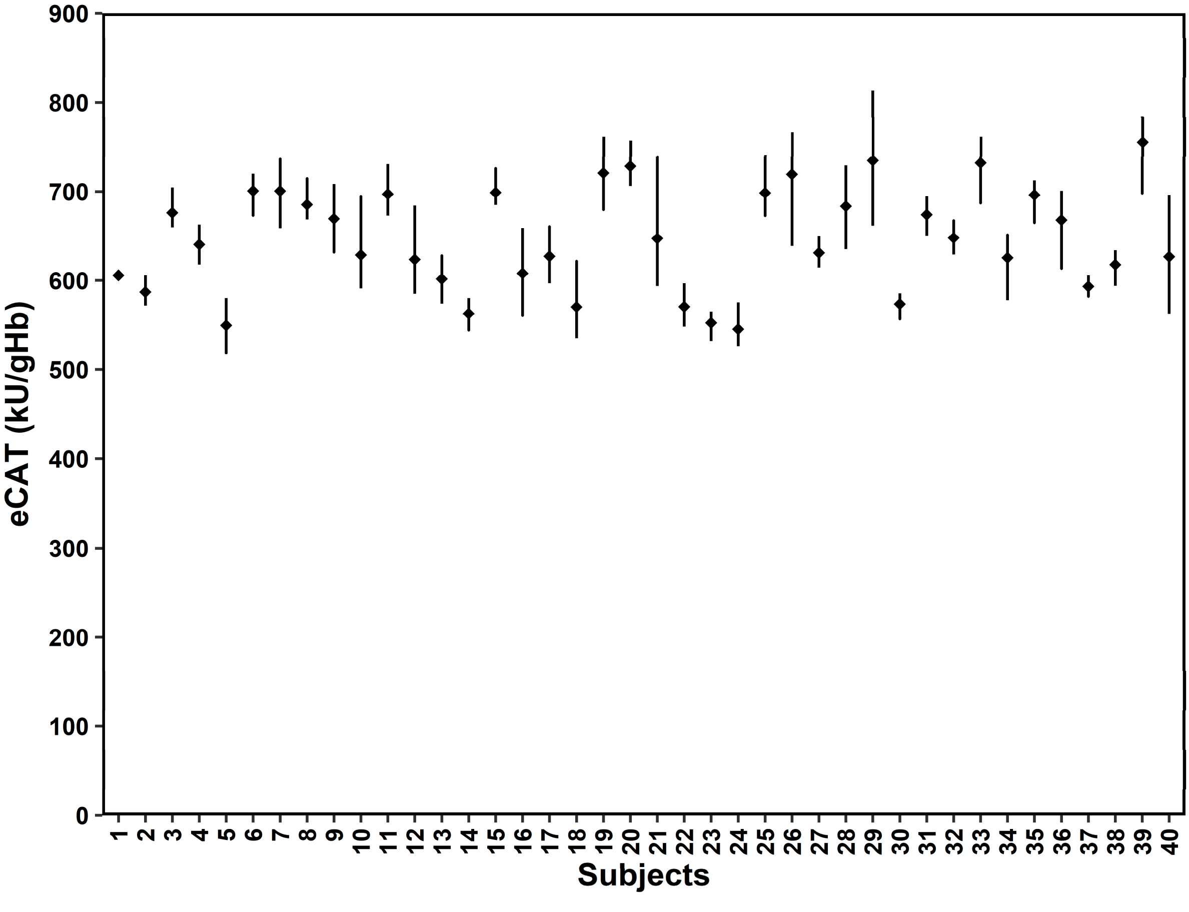

Intra-individual variation and reliability of biomarkers of the antioxidant defense system by considering dietary and lifestyle factors in premenopausal women

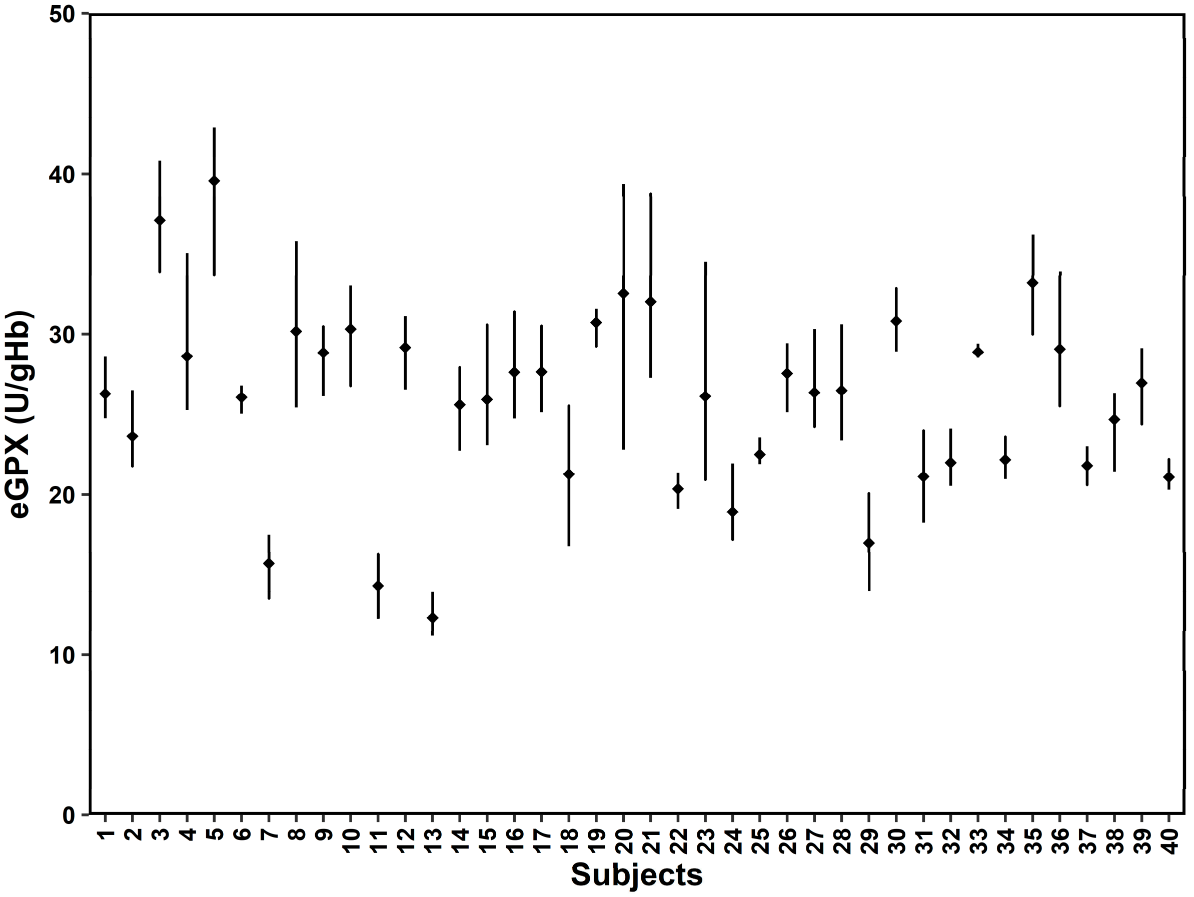

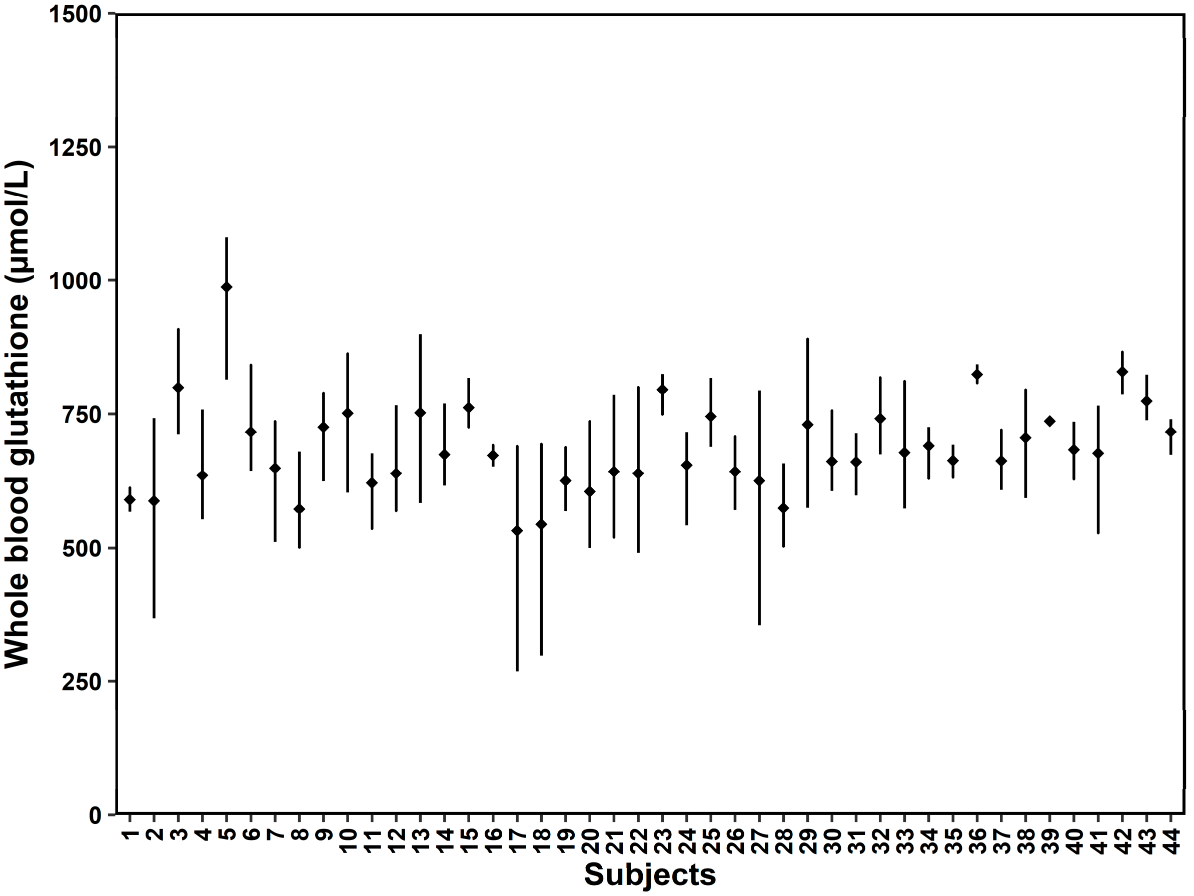

Intra-individual variation and reliability of biomarkers of the antioxidant defense system by considering dietary and lifestyle factors in premenopausal women

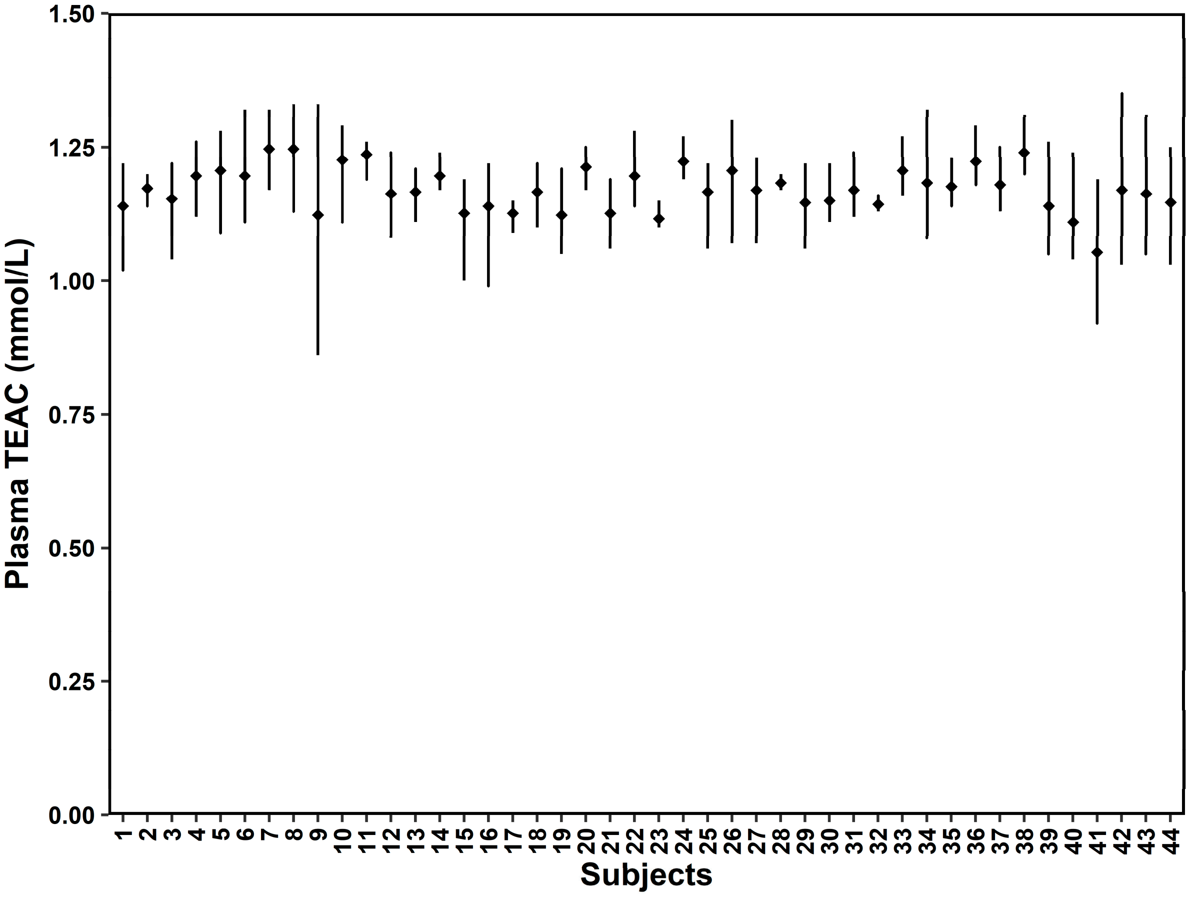

Supplementary Figure S3 Mean values (filled rhombus) and absolute ranges (error bar) of antioxidant biomarkers for each subject over the study period

Abbreviations: eCAT, erythrocyte catalase; eGPx, erythrocyte glutathione peroxidase; TEAC, Trolox equivalent antioxidant capacity.

Supplementary Table S1 Crude and adjusted indicators of reliability and inter- and intra-individual variability for each antioxidant biomarker in premenopausal women after excluding subjects with outlying residuals [a, b]

	n [c]	ICC_{unadj} [95% CI]	CV_G	CV_T	II	$RCV_{\%,pos}$	$RCV_{\%,neg}$	$n_{\beta,10\ \%}$	$n_{\beta,20\ \%}$	*n* [c]	ICC_{adj} [95% CI] [d]
Serum bilirubin (μmol/L)	40	0.604 [0.428, 0.735]	0.344	0.279	0.809	±77.3		6	3	40	0.587 [0.420, 0.754]
Serum uric acid (μmol/L) [e]	41	0.746 [0.609, 0.838]	0.176	0.102	0.580	+32.5	-24.5	3	2	36	0.816 [0.712, 0.904]
Plasma vitamin C (μmol/L) [e]	39	0.563 [0.371, 0.710]	0.123	0.108	0.880	+34.7	-25.8	7	3	42	0.578 [0.405, 0.743]
Plasma α-tocopherol (μmol/L) [e]	42	0.752 [0.611, 0.843]	0.175	0.100	0.571	+31.7	-24.1	3	2	41	0.577 [0.409, 0.744]
Plasma γ-tocopherol (nmol/L) [e]	34	0.841 [0.725, 0.904]	0.466	0.195	0.418	+70.7	-41.4	2	1	33	0.826 [0.728, 0.914]
Plasma retinol (nmol/L) [e]	43	0.535 [0.330, 0.682]	0.193	0.180	0.931	+64.0	-39.0	8	4	43	0.435 [0.239, 0.633]
Plasma α-carotene (nmol/L) [e]	36	0.879 [0.794, 0.930]	0.558	0.195	0.349	+70.7	-41.4	2	1	35	0.883 [0.819, 0.941]
Plasma β-carotene (nmol/L) [e]	34	0.939 [0.891, 0.965]	0.562	0.134	0.238	+44.6	-30.8	1	1	34	0.901 [0.840, 0.950]
Plasma lutein (nmol/L) [e]	43	0.681 [0.509, 0.792]	0.336	0.227	0.675	+86.1	-46.3	5	2	38	0.423 [0.224, 0.649]
Plasma zeaxanthin (nmol/L) [e]	32	0.801 [0.666, 0.883]	0.396	0.192	0.484	+69.3	-40.9	3	1	38	0.558 [0.388, 0.742]
Plasma β-cryptoxanthin (nmol/L) [e]	34	0.878 [0.784, 0.927]	0.399	0.144	0.361	+48.8	-32.8	2	1	36	0.869 [0.791, 0.931]
Plasma lycopene (nmol/L) [e]	42	0.737 [0.589, 0.833]	0.399	0.233	0.583	+88.9	-47.1	4	2	41	0.533 [0.363, 0.715]
Serum coenzyme Q10 (nmol/L) [e]	43	0.767 [0.627, 0.852]	0.292	0.159	0.543	+54.8	-35.4	3	2	41	0.572 [0.407, 0.744]
eCAT (kU/gHb)	38	0.699 [0.542, 0.813]	0.084	0.055	0.656	±15.4		4	2	37	0.642 [0.484, 0.802]
eGPx (U/gHb) [e]	37	0.822 [0.709, 0.892]	0.245	0.113	0.460	+36.5	-26.8	2	1	37	0.854 [0.773, 0.924]
Blood glutathione (μmol/L)	36	0.247 [0.025, 0.448]	0.069	0.120	1.748	±33.2		28	13	36	0.106 [0.000, 0.369]
Plasma TEAC (mmol/L) [f]	36	0.000 [0.000, 0.189]	0.000	0.061	/	±17.0		/	/	37	0.000 [0.000, 0.249]

Abbreviations: ICC, intraclass correlation coefficient; CV_G, inter-individual variation; CV_T, total intra-individual variation; II, index of individuality; RCV, reference change value; $n_{\beta,\ 10\ \%}$, number of required measurements to limit the attenuation in regression coefficient to 10 %; $n_{\beta,\ 20\ \%}$, number of required measurements to limit the attenuation in regression coefficient to 20 %; eCAT, erythrocyte catalase; Hb, hemoglobin; eGPx, erythrocyte glutathione peroxidase; TEAC, Trolox equivalent antioxidant capacity.

[a] For each considered subject, three mean values of the respective blood biomarker were available.

[b] Except for the adjusted ICC, variance components were estimated by restricted maximum likelihood method via one-way random effect model.

[c] Sample size after excluding pre-identified outliers by Cochran's test and Reed's criterion as well as subjects with at least one model-wise outlying residuum (i.e., > 2.0) of the applied model. In addition, in the calculation of the adjusted ICC, the sample size was reduced by one subject because one subject provided no dietary record in the third assessment.

[d] Adjusted ICC was calculated using the variance components estimated by restricted maximum likelihood method via a linear mixed-effects model including the following fixed effects: time elapsed since the first day of the last menstrual period (d), body mass index (kg/m²), fasting duration (h), sleep duration of the last night (h), use of contraceptives (no vs. yes), serum cholesterol concentration (mmol/L) and physical activity level. In addition, energy intake (MJ/d) or, if applicable, nutrient intake levels (i.e. purine intake (mg/d) for serum uric acid, vitamin C intake (mg/d) for plasma vitamin C, tocopherol equivalents (mg/d) for both plasma tocopherol parameters, retinol equivalents (mg/d) for plasma retinol and β-carotene (mg/d) for plasma carotenoids) were included in the model, respectively.

[e] Biomarker values were logarithmically transformed (natural logarithm).

[f] Model with a singular fit.
